# GOexpress: an R/Bioconductor package for the identification and visualisation of robust gene ontology signatures through supervised learning of gene expression data

**DOI:** 10.1186/s12859-016-0971-3

**Published:** 2016-03-11

**Authors:** Kévin Rue-Albrecht, Paul A. McGettigan, Belinda Hernández, Nicolas C. Nalpas, David A. Magee, Andrew C. Parnell, Stephen V. Gordon, David E. MacHugh

**Affiliations:** Animal Genomics Laboratory, UCD School of Agriculture and Food Science, University College Dublin, Dublin 4, Ireland; Centre for Pharmacology and Therapeutics, Division of Experimental Medicine, Imperial College London, Hammersmith Hospital, London, W12 0NN UK; Novartis Pharmaceuticals, Elm Park Business Campus, Merrion Road, Dublin 4, Ireland; UCD School of Mathematics and Statistics, Insight Centre for Data Analytics, University College Dublin, Dublin 4, Ireland; Proteome Center Tübingen, Interfaculty Institute for Cell Biology, University of Tübingen, Auf der Morgenstelle 15, 72076 Tübingen, Germany; UCD School of Veterinary Medicine, University College Dublin, Dublin 4, Ireland; UCD Conway Institute of Biomolecular and Biomedical Research, University College Dublin, Dublin 4, Ireland

**Keywords:** Gene expression, Gene ontology, Supervised learning, Classification, Microarray, RNA-sequencing, Functional genomics

## Abstract

**Background:**

Identification of gene expression profiles that differentiate experimental groups is critical for discovery and analysis of key molecular pathways and also for selection of robust diagnostic or prognostic biomarkers. While integration of differential expression statistics has been used to refine gene set enrichment analyses, such approaches are typically limited to single gene lists resulting from simple two-group comparisons or time-series analyses. In contrast, functional class scoring and machine learning approaches provide powerful alternative methods to leverage molecular measurements for pathway analyses, and to compare continuous and multi-level categorical factors.

**Results:**

We introduce GOexpress, a software package for scoring and summarising the capacity of gene ontology features to simultaneously classify samples from multiple experimental groups. GOexpress integrates normalised gene expression data (e.g., from microarray and RNA-seq experiments) and phenotypic information of individual samples with gene ontology annotations to derive a ranking of genes and gene ontology terms using a supervised learning approach. The default random forest algorithm allows interactions between all experimental factors, and competitive scoring of expressed genes to evaluate their relative importance in classifying predefined groups of samples.

**Conclusions:**

GOexpress enables rapid identification and visualisation of ontology-related gene panels that robustly classify groups of samples and supports both categorical (e.g., infection status, treatment) and continuous (e.g., time-series, drug concentrations) experimental factors. The use of standard Bioconductor extension packages and publicly available gene ontology annotations facilitates straightforward integration of GOexpress within existing computational biology pipelines.

**Electronic supplementary material:**

The online version of this article (doi:10.1186/s12859-016-0971-3) contains supplementary material, which is available to authorized users.

## Background

Following the rapid decrease in the cost of high-throughput sequencing and the standardisation of analytical pipelines for microarray data, complex multifactorial experimental designs have become commonplace in many research fields. Many different methodologies have been proposed to address data summarisation and visualisation at the pathway level [1–3]. Gene ontology (GO) is one of the most robust and widely used resources to categorise biological entities into functionally related groups [4–6]. The most common system biology techniques currently used to extract biological knowledge from transcriptomics data sets often apply gene-set enrichment analysis (GSEA) on gene lists resulting from differential expression outputs. However, this approach has two limitations: (1) GSEA approaches are typically limited to the analysis of a single gene list resulting from a two-group comparison, and (2) gene expression estimates in the individual replicates are lost in differential expression statistics, potentially obscuring outliers. The first limitation can be circumvented by combining multiple comparisons into a summary statistic assigned to each gene feature (e.g., time-series). To the best of our knowledge no publicly available tool addresses the second limitation and provides a simple interface to access and visualise individual gene expression profiles following the identification of relevant genes and molecular pathways. The GOexpress software package described here provides a number of functions for visualisation of gene expression data from multi-factorial experimental designs, both as individual gene profiles or summarised as functionally-related gene sets. Additionally, the package facilitates the use of supervised classification or parametric analysis of variance, which complement existing approaches to identify gene features that best discriminate multiple groups of samples. Indeed, while parametric differential expression approaches are widely used to identify significant changes in expression levels, non-parametric supervised learning and classification methods represent a valuable alternative strategy to identify modest yet consistent differences, even between limited numbers of replicates [7, 8].

Although the underlying technologies are very different, RNA-sequencing (RNA-seq) and microarray transcriptomic approaches both yield quantitative expression levels for each gene in each sample. Typically, this expression matrix is filtered to retain only ‘informative’ genes (e.g., > 1 read count per million [CPM] in at least *n* biological replicates for RNA-seq). In addition, the removal of genes expressed at very low levels is normally performed to minimise stochastic background expression at the lower ends of the dynamic ranges for both RNA-seq and microarray technologies [9–11]. Traditionally, differential expression analysis of transcriptomics data sets has been performed using parametric statistical methods such as edgeR [12] or limma [13]. However, non-parametric methods using bootstrapping (e.g., IsoDE) or non-parametric empirical Bayesian-based approaches (e.g., GFOLD) have been shown to perform equally well or better using transcriptomics data sets with few or no replicates to produce biologically meaningful rankings of differentially expressed genes [14, 15].

The non-parametric random forest (RF) algorithm has been shown to perform comparably or better than other methods for both microarray and RNA-seq data sets [16, 17]. It shows excellent performance even with high levels of noise; it is a powerful method for feature selection (e.g., identification of biomarkers); it can be used when the number of variables is significantly larger than the number of observations; and for data sets involving two or more experimental groups [18–20]. Moreover, in contrast to parametric approaches intended to optimally detect differences in mean expression among groups, the non-parametric RF algorithm is assumption-free regarding parameters of the distributions underlying gene expression patterns (i.e., mean, variance), facilitating detection of more subtle associations between gene expression levels and phenotypes [21].

In this paper we extend the RF approach for gene feature scoring by integrating a supervised RF analysis with a subsequent summarisation at the GO level, to identify robust panels of functionally related genes that best classify multiple sample groups. This multi-classifier fusion approach differs from other functional class scoring (FCS) methods: it combines classification of multiple groups with ranking information to identify GO terms that best classify samples, and estimates probability of GO ranking through permutation-based *P*-values. It is important to emphasise that GOexpress is not a conventional GSEA tool; its purpose is to use supervised learning to prioritize gene sets within GO functional classes that best classify samples according to their known experimental labels. In addition, the non-parametric RF algorithm has many desirable features, including considerable robustness to outliers, and absence of overfitting [21, 22]. The software implementation provides a range of visualisation functions, and seamless integration with the R/Bioconductor framework. It also reduces the burden of programming for non-expert users, while providing a route to more advanced applications (e.g., R/Shiny).

To demonstrate the capabilities of the GOexpress package we use a previously analysed and published multifactorial microarray gene expression data set [23]. A separate demonstration RNA-seq data set is also included with the package and the package vignette guides users through the implementation of the corresponding analysis workflow, which is essentially identical to that of microarray data provided in Additional file 1.

## Implementation

### General aspects and design

**Fig. 1:**
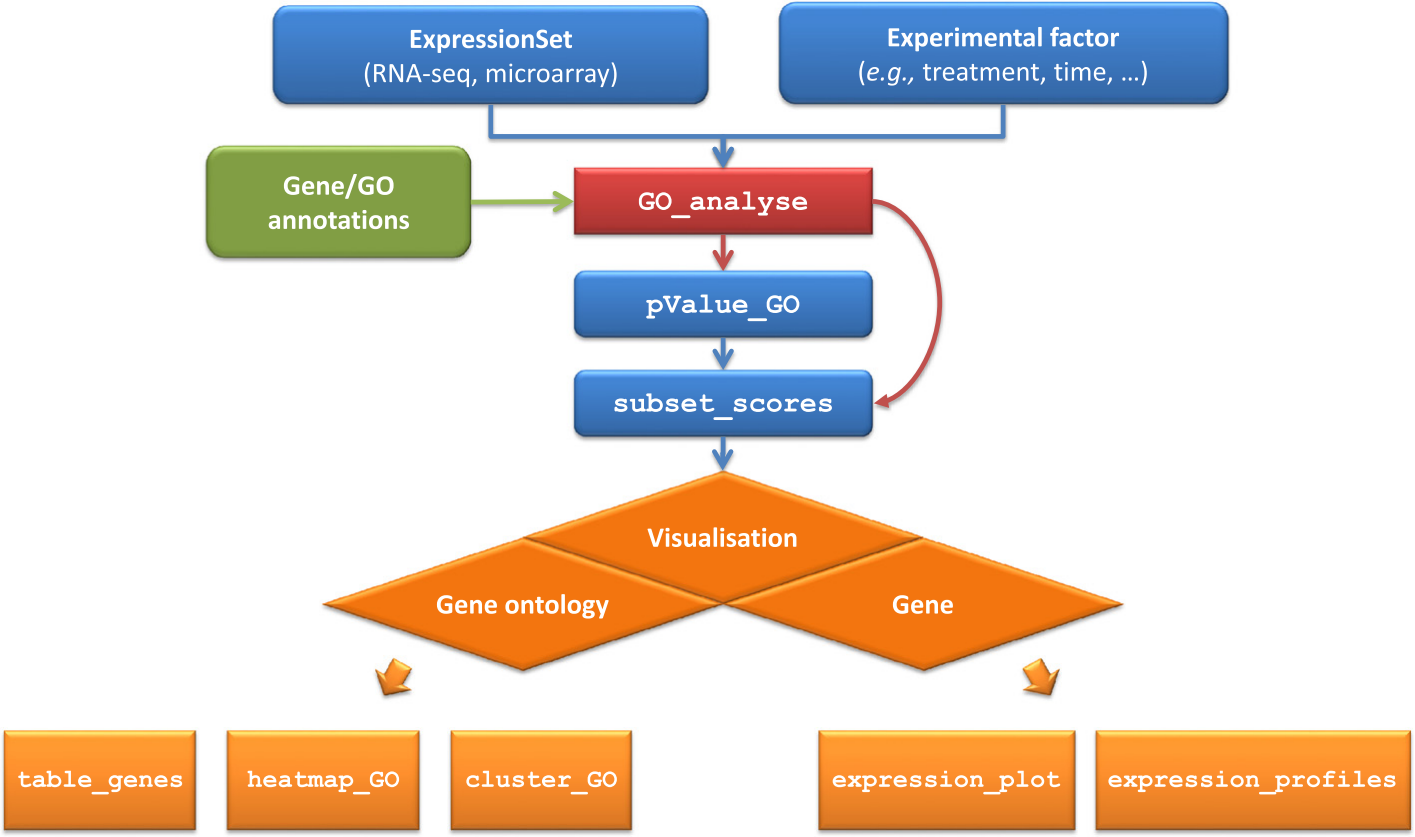
Overview of the GOexpress workflow. A typical GOexpress analysis takes as input: an ExpressionSet of the Biobase Bioconductor package containing either microarray or RNA-seq normalised expression data; the name of an experimental factor present in the phenoData slot of the ExpressionSet; and annotations for the features and GO terms (or other functional classes) considered. The GO_analyse function calculates scores and ranks for the individual genes and GO terms. Optionally, the pValue_GO function randomly permutes the gene features to estimate the probability of each GO term to rank (or score) higher by chance. Finally, various functions allow visualisation of gene expression profiles by gene and gene ontology, and export of the calculated statistics in text files

GOexpress is written entirely in the R programming language [24] and relies on several other widely used R packages available from Bioconductor [25, 26] (biomaRt [27, 28]) and CRAN packages (ggplot2 [29], randomForest [30], RColorBrewer, stringr, VennDiagram). The entry point for the package is the function GO_analyse, which processes the input gene expression data and returns the resulting scoring tables and annotations required for all downstream filtering and visualisation functions (Fig. 1). Critically, GOexpress does not transform the input expression data in any way. For the input ExpressionSet, GOexpress is designed to use normalised expression data pre-processed using widely available dedicated software packages; for example, log_2_-transformed microarray intensities normalised using the farms package or log_2_-transformed counts per million obtained using the edgeR package for RNA-seq data. Conveniently, the function allows analyses based on a subset of samples defined on-the-fly, without the need to manually create multiple input ExpressionSet objects. The procedure performed by the GO_analyse function comprises several steps: (1) collection of gene and gene ontology annotations, (2) evaluation and ranking of genes by capacity to classify groups of samples, (3) summarisation of classification power at the GO level, and (4) formatting of resulting statistics into a structured list returned to the user (Fig. 1). These steps are described in more detail in the following sections.

### Semi-automated annotation of input gene expression data

The GO_analyse function requires a minimum of two mandatory user inputs. The package source archive includes an example of each input and output (Additional file 2). Firstly, the function expects pre-processed expression data (i.e., filtered and normalised), and associated sample phenotypic information loaded in the assayData and phenoData containers of an ExpressionSet object [Bioconductor Biobase package] (Fig. 1). This standardised format ensures interoperability with other Bioconductor packages, and simplifies data handling. The second mandatory user input is the name of an experimental factor—with two or more levels—present in phenoData. The function will consequently estimate the capacity of each gene and GO term to classify groups of samples associated with different levels of that experimental factor based on the provided expression data.

In many cases these two arguments are sufficient due to retrieval of gene and GO annotations from the current Ensembl release using the Bioconductor biomaRt package. However, it is strongly recommended to generate a local copy of all annotations for two key reasons: (1) to ensure traceability and reproducibility of results, even when new Ensembl annotations are released; and (2) to avoid multiple calls to the web Ensembl BioMart API, saving significant runtime during the execution of the function. Additionally, custom annotations may also be provided to analyse datasets using gene feature identifiers not currently supported (Fig. 1).

The term “gene feature” will henceforth refer to either microarray probeset identifiers or Ensembl gene identifiers, the two types of feature identifiers supported by the automated annotation procedure. Custom annotations are imported in three independent data frames: (1) a two-column table that maps gene features to GO terms, (2) individual gene annotations that include the gene ID and associated gene name with an optional short description, and (3) individual GO annotations that include the corresponding name (e.g., “catalytic activity”) and namespace (e.g., “molecular function”). The mapping table must also include genes absent from the expression data set such that all known annotated genes are used as a background set for the scoring of individual genes and GO terms. Genes present in the annotations, but absent from the expression data set, will be assigned a score of 0 and a corresponding rank equal to the number of genes in the expression data set plus one, impacting the subsequent scoring and ranking of their associated GO terms, if any. This choice is further discussed below for the scoring of GO terms.

### Scoring of genes using expression data

Currently, the RF algorithm is used as the default method to answer the question: “*How well does each gene feature in the expression data set discriminate between multiple groups of samples?*” The RF algorithm consists of multiple decision trees; each internal node in each classification tree is built based on a different bootstrap sample (with replacement) of observations (i.e., biological samples) and a random sample of variables (i.e., gene features). The randomForest package first calculates the Gini index [31] for each node in the tree, where the Gini index is a measure of homogeneity from 0 (homogeneous) to 1 (heterogeneous). The decrease in Gini index resulting from a split on a variable is then calculated for each node, and averaged for each variable over all the trees in the model. The variable with the largest mean decrease in the Gini index is then considered the most important. Technically, GOexpress uses the mean decrease in Gini index as the score for each gene feature in the expression data set.

A key feature of the RF approach is the implicit handling of interactions between genes. In order to provide a robust solution, each tree in the random forest is built on a bootstrap sample of observations. As the trees are grown, a random sample of genes is selected for each internal node and these genes are tested for their individual capacity to improve the partitioning reached in the previous node. The larger the number of trees built, the more complete the coverage of interactions between gene features.

### Summarisation and ranking of GO terms

**Fig. 2:**
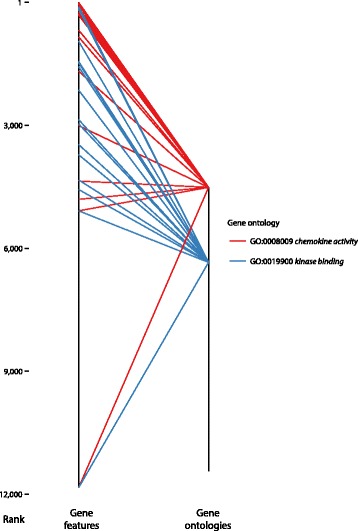
Ranking of filtered GO terms by summarisation of gene ranks. The rank of each gene feature is shown on the left, while the average rank of each GO term (average of all annotated genes) is shown on the right. The ranks of all genes associated with the 1st- and 55th-ranked GO terms are shown, following filtering for only molecular function GO terms associated with at least 15 genes in the annotations. Notably, eight and 13 genes associated with the GO terms *chemokine activity* and *kinase binding* are absent from the sample ExpressionSet and ranked last

The GO_analyse function scores each GO term by aggregating the rank (or alternatively, the score) for all annotated genes associated with the term obtained in the previous step (Fig. 2, Additional file 3).

All genes annotated to GO terms but absent from the expression data set are considered to have no classification power, and are assigned a score of 0 and the worst rank preserving the continuity of ranking. This procedure is particularly suited to transcriptomics data sets where uninformative gene features (e.g., below a detection threshold) are filtered prior to the analysis. Indeed, during the summarisation step, these uninformative genes negatively impact the ranking of corresponding GO terms. Consequently, this summarisation approach implicitly favours GO terms over-represented in the data set, in addition to integrating the ranking information for genes present in the ExpressionSet.

### Formatting of results for downstream analyses

The final step of the GO_analyse function is to assemble the resulting tables of ranked genes and GO terms with important analysis parameters for traceability and reproducibility. These include the subset of samples used for the analysis (if applicable), the number of decision trees and the number of gene features sampled (if the default RF algorithm was used), and the summarisation function used to score each GO term based on the scores of all their associated gene features. Alternatively, one-way analysis of variance (ANOVA) is available as a parametric statistical scoring approach.

## Results

### Sample data

We demonstrate the use of GOexpress using microarray data from a previously published and well-characterised multifactorial microarray gene expression data set [23, 32–36]. In this example, monocyte-derived macrophage (MDM) transcriptomes from five different cattle were examined over three time points spanning a 24-hour time course, following one of each of the following four treatments: (1) infection with *Mycobacterium bovis*, (2) infection with *M. bovis* BCG, (3) infection with *M. avium* subsp. *paratuberculosis*, and (4) culture media for non-infected controls [33]. The ExpressionSet used here was obtained following normalisation and filtering of informative probesets using the Bioconductor farms package [37], leaving 11,842 probesets measured in 65 samples (Additional file 4).

### Probeset-level classification and visualisation

In the example below, we use GOexpress to identify and visualise the genes and GO terms that best classify samples subjected to the four different treatments across all time points post-infection. Given that the ExpressionSet also includes control samples prior to infection, we use the subset argument of the GO_analyse function to consider only the samples post-infection (i.e., 2, 6, and 24 h post-infection). We also use local versions of genome-wide annotations for the Affymetrix® GeneChip® Bovine Genome Array downloaded from the Ensembl release 77 (Additional file 1).
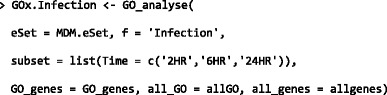


**Fig. 3:**
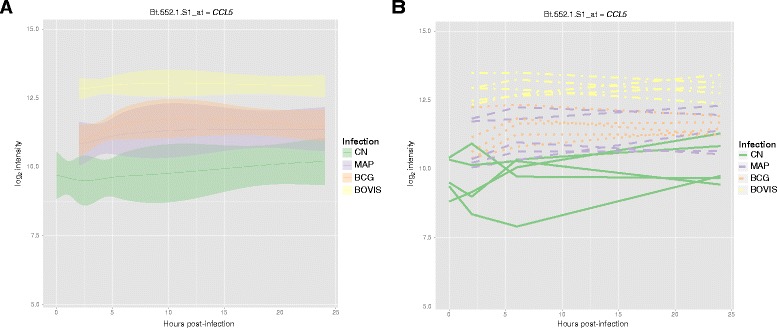
Expression profiles for the top-ranked microarray probeset that best clusters treatment groups. The expression_plot and expression_profiles visualisation functions facilitate summarisation of gene expression levels for *CCL5* by sample group (**a**), or individual sample series (**b**) for each experimental infection (*green*: uninfected MDM; *purple*: *M. avium* subspecies *paratuberculosis*; *orange*: *M. bovis* BCG; *yellow*: *M. bovis*)

In the resulting object, the table of ranked genes (Additional file 5) demonstrates that the probeset Bt.552.1.S1_at (gene name *CCL5*), best classifies the samples according to treatment group. It is also possible to use the expression_plot and expression_profiles family of functions to visualise group trends and individual gene expression profiles for the four treatment groups (Fig. 3).

To assess the performance of the RF algorithm we compared the importance score—i.e., the decrease in the Gini index—to the *F* ratio obtained using the one-way ANOVA method also implemented in GOexpress, considering the same four treatment groups (Additional file 6). The two methods show good agreement, with a positive Pearson correlation coefficient of 0.69 (*P*-value < 2.2 × 10^−16^). In particular, the best (i.e., top ranked) classifiers identified by the RF tend to display a high variance among groups relative to the variance within groups. Conversely, poor classifiers identified by the RF generally display a low *F* ratio. Notably, the RF produces generally more conservative results; indeed, a number of features identified as significant using the one-way ANOVA (FDR < 0.05) show little or no classification power (Additional file 6).

### Ontology-level summarisation

#### Permutation-based *P*-value

From an ontology perspective, the basic GO results present two limitations. Firstly, all three types of ontology—biological process, molecular function, and cellular component—are all merged in a single table. Secondly, and most importantly, GO terms associated with fewer genes are favoured at the top of the ranking. This happens because the highest possible average rank for a group of five genes is 3, as opposed to 3,000 for a group of 6,000 genes. Additionally, GO terms associated with small numbers of genes are more susceptible to outliers and single gene effects in the expression data; this problem is not normally observed for GO terms associated with larger numbers of genes. Moreover, when small GO terms are filtered out, this scoring method emphasises specific and well-defined GO terms (e.g., GO:0070233: *negative regulation of T cell apoptotic process*), as opposed to the broad higher-level and generally less informative GO terms (e.g., GO:0005515: *protein binding*). Importantly, the effect of gene ontology size is an acknowledged issue of pathway analysis, most methods focusing the analysis on pathways that pass specific size thresholds [38].

To assess the probability of GO term ranking, GOexpress includes the function pValue_GO that randomises the gene feature ranking table, and produces a permutation-derived *P*-value that indicates the proportion of permutations where each GO term is ranked equal or higher relative to the original result (Additional file 7). In other words, these *P*-values directly translate into the estimated probability of each set of functionally-related genes reaching their calculated average rank by chance. In addition, it is important to note that *P*-values obtained for each GO term in this manner are clearly non-independent, due to the competitive ranking of genes and subsequent GO term summarisation. Therefore, *P*-value adjustment for multiple testing is not recommended in this case.
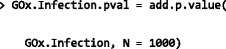


To assess the performance of the GO summarisation step, we compared our results to those obtained using the widely used GSA package [39] (Additional file 8). In a similar fashion to GOexpress, GSA determines the significance of pre-defined sets of genes with respect to an outcome variable, such as a multiclass group indicator. The “maxmean” test implemented in GSA was designed to detect unusually large and coordinated gene scores indicative of differentially expressed gene sets; significant gene sets are identified as those where most of the genes show either higher or lower expression correlating with the group indicator. In contrast, the RF approach implemented in GOexpress identifies gene sets containing both up- and down-regulated genes that, together, contribute to improve the classification of samples into their respective phenotypic groups. Notably, the supervised classification approach implemented in the RF algorithm was previously shown to have superior power to detect association of gene expression level with phenotype relative to a traditional Significance Analysis of Microarray extended to Gene-Set analyses (SAM-GS) in the presence of correlations between gene expression profiles, with similar performance in the absence of correlated gene expression [21].

Comparison of the average-rank and permutation-based *P*-value implemented in GOexpress to the GSA approach, revealed that four of the seven GO terms identified by GSA (FDR < 0.05) were also found to be significant (*P*-value < 0.05) using GOexpress. Interestingly, all seven GO terms are biological processes. In contrast, GOexpress returned an additional 18 GO terms (14 biological processes [BP], three cellular components [CC], four molecular functions [MF]); these include additional relevant functional categories such as *positive regulation of NF-kappaB transcription factor activity* (BP), *chemokine activity* (MF), and *immunological synapse* (CC) [Additional file 8]. Notably, the largest average-score metric available in GOexpress also emphasises functional categories that are biologically highly relevant, including chemoattractant activity such as *cellular response to interleukin-1* (BP) and *chemoattractant activity* (MF) [Additional file 8]. Taken together, those results indicate that GOexpress detects additional functional categories capable of improving the classification of samples, while GSA may be restricted yet more sensitive in detecting coordinated expression changes within gene sets [39].

#### Filtering and visualisation

Using the subset_scores filtering function, users may filter GO terms according to domain (i.e., biological process, molecular function or cellular component), and/or minimal count of annotated gene features. In the example shown below, only GO terms associated with at least 15 genes, and an empirical *P*-value ≤ 0.05 are retained.
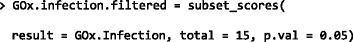


The resulting object is a list with the same structure as the input object and an additional element stating the filtering criteria applied. In this example, the molecular function *chemokine activity* (GO:0008009), which is associated with 35 genes in the annotations—27 of which are present in the ExpressionSet—is ranked as the top GO term that best classifies the four treatment groups across all time points (Additional file 7). Those results are consistent with our previously published finding highlighting the role of chemokine signalling and communication between innate and adaptive immune cells in the differential response to virulent and attenuated mycobacterial infections [23].

**Fig. 4:**
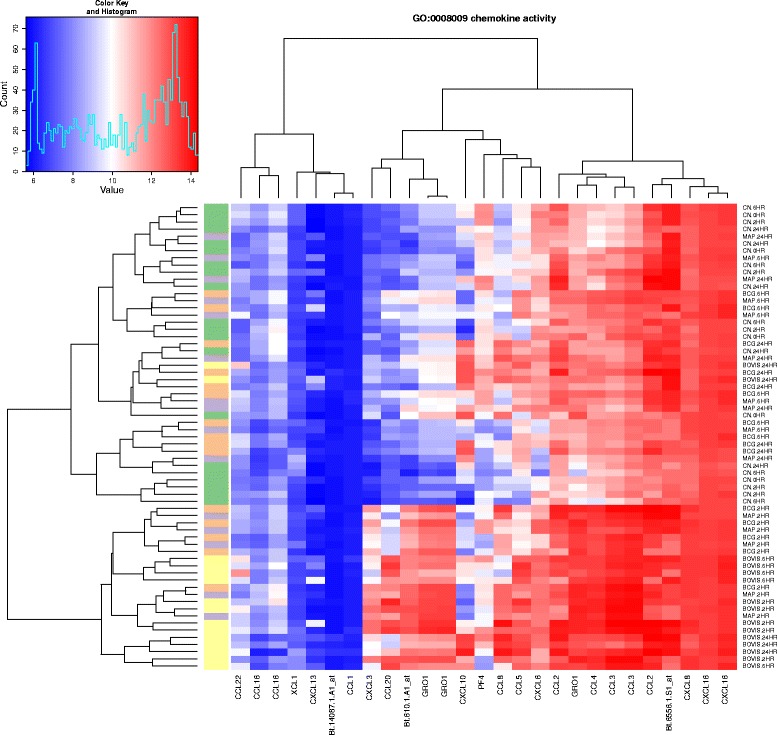
Heat map and hierarchical clustering of treatment groups using expression data from genes associated with the top-ranked GO term. The heatmap_GO visualisation function summarises expression level for all genes present in the ExpressionSet and associated with *chemokine activity* (GO:0008009). *Green*: uninfected MDM; *purple*: *M. avium* subspecies *paratuberculosis*; *orange*: *M. bovis* BCG; *yellow*: *M. bovis*

Following this, using the heatmap_GO function, it is possible to visualise the expression level of all genes associated with the GO term in each sample, as well as the hierarchical clustering of samples and probesets resulting from the corresponding expression profiles (Fig. 4). Additionally, the table_genes function allows users to export individual scores, ranks and annotations for genes associated with a particular GO term (Table 1).

**Table 1:** Feature-level statistics for the microarray probesets associated with the top-ranked GO term

Probeset	Score	Rank	Gene name	Description
Bt.552.1.S1_at	0.356	1	*CCL5*	Bos taurus chemokine (C-C motif) ligand 5 (CCL5), mRNA. [Source:RefSeq mRNA;Acc:NM_175827]
Bt.28088.1.S1_at	0.121	33	*CXCL13*	Bos taurus chemokine (C-X-C motif) ligand 13 (CXCL13), mRNA. [Source:RefSeq mRNA;Acc:NM_001015576]
Bt.22009.1.S1_at	0.114	38	*CXCL16*	Bos taurus chemokine (C-X-C motif) ligand 16 (CXCL16), mRNA. [Source:RefSeq mRNA;Acc:NM_001046095]
Bt.9560.1.S1_at	0.097	57	*CCL20*	Bos taurus chemokine (C-C motif) ligand 20 (CCL20), mRNA. [Source:RefSeq mRNA;Acc:NM_174263]
Bt.23093.1.S1_at	0.049	130	*CXCL3*	Bos taurus chemokine (C-X-C motif) ligand 3 (CXCL3), mRNA. [Source:RefSeq mRNA;Acc:NM_001046513]
Bt.611.1.S1_at	0.037	166	*GRO1*	Bos taurus chemokine (C-X-C motif) ligand 1 (melanoma growth stimulating activity, alpha) (GRO1), mRNA. [Source:RefSeq mRNA;Acc:NM_175700]
Bt.611.1.S1_x_at	0.033	192	*GRO1*	Bos taurus chemokine (C-X-C motif) ligand 1 (melanoma growth stimulating activity, alpha) (GRO1), mRNA. [Source:RefSeq mRNA;Acc:NM_175700]
Bt.9504.1.A1_at	0.028	244	*CCL4*	Bos taurus chemokine (C-C motif) ligand 4 (CCL4), mRNA. [Source:RefSeq mRNA;Acc:NM_001075147]
Bt.611.1.S2_at	0.025	277	*GRO1*	Bos taurus chemokine (C-X-C motif) ligand 1 (melanoma growth stimulating activity, alpha) (GRO1), mRNA. [Source:RefSeq mRNA;Acc:NM_175700]
Bt.22009.2.S1_a_at	0.023	298	*CXCL16*	Bos taurus chemokine (C-X-C motif) ligand 16 (CXCL16), mRNA. [Source:RefSeq mRNA;Acc:NM_001046095]
Bt.21088.1.S1_at	0.023	301	*CCL22*	Bos taurus chemokine (C-C motif) ligand 22 (CCL22), mRNA. [Source:RefSeq mRNA;Acc:NM_001099162]
Bt.14087.1.A1_at	0.023	307		Uncharacterized protein [Source:UniProtKB/TrEMBL;Acc:E1BGB8]
Bt.2408.1.S1_at	0.013	677	*CCL2*	Bos taurus chemokine (C-C motif) ligand 2 (CCL2), mRNA. [Source:RefSeq mRNA;Acc:NM_174006]
Bt.154.1.S1_at	0.011	844	*CCL8*	Bos taurus chemokine (C-C motif) ligand 8 (CCL8), mRNA. [Source:RefSeq mRNA;Acc:NM_174007]
Bt.8144.1.S1_at	0.006	1679	*XCL1*	Bos taurus chemokine (C motif) ligand 1 (XCL1), mRNA. [Source:RefSeq mRNA;Acc:NM_175716]
Bt.7165.1.S1_at	0.003	3007	*CXCL6*	Bos taurus chemokine (C-X-C motif) ligand 6 (granulocyte chemotactic protein 2) (CXCL6), mRNA. [Source:RefSeq mRNA;Acc:NM_174300]
Bt.610.1.A1_at	0.002	4360		Bos taurus chemokine (C-X-C motif) ligand 2 (CXCL2), mRNA. [Source:RefSeq mRNA;Acc:NM_174299]
Bt.9974.1.S1_at	0.001	4805	*CCL3*	Bos taurus chemokine (C-C motif) ligand 3 (CCL3), mRNA. [Source:RefSeq mRNA;Acc:NM_174511]
Bt.9974.1.S1_a_at	0.000	5086	*CCL3*	Bos taurus chemokine (C-C motif) ligand 3 (CCL3), mRNA. [Source:RefSeq mRNA;Acc:NM_174511]
Bt.6556.1.S1_at	0.000	5086		Bos taurus regakine 1 (LOC504773), mRNA. [Source:RefSeq mRNA;Acc:NM_001034220]
Bt.21950.1.S1_at	0.000	5086	*CCL16*	chemokine (C-C motif) ligand 16 [Source:HGNC Symbol;Acc:HGNC:10614]
Bt.21950.1.S1_s_at	0.000	5086	*CCL16*	chemokine (C-C motif) ligand 16 [Source:HGNC Symbol;Acc:HGNC:10614]
Bt.20673.1.A1_at	0.000	5086	*CCL1*	chemokine (C-C motif) ligand 1 [Source:HGNC Symbol;Acc:HGNC:10609]
Bt.2408.1.S1_s_at	0.000	5086	*CCL2*	Bos taurus chemokine (C-C motif) ligand 2 (CCL2), mRNA. [Source:RefSeq mRNA;Acc:NM_174006]
Bt.155.1.S1_at	0.000	5086	*CXCL8*	Bos taurus interleukin 8 (IL8), mRNA. [Source:RefSeq mRNA;Acc:NM_173925]
Bt.11581.1.S1_at	0.000	5086	*PF4*	Bos taurus platelet factor 4 (PF4), mRNA. [Source:RefSeq mRNA;Acc:NM_001101062]
Bt.16966.1.S1_at	0.000	5086	*CXCL10*	Bos taurus chemokine (C-X-C motif) ligand 10 (CXCL10), mRNA. [Source:RefSeq mRNA;Acc:NM_001046551]

The table_genes function was used to export results for the top-ranked GO term *chemokine activity* (GO:0008009)

**Fig. 5:**
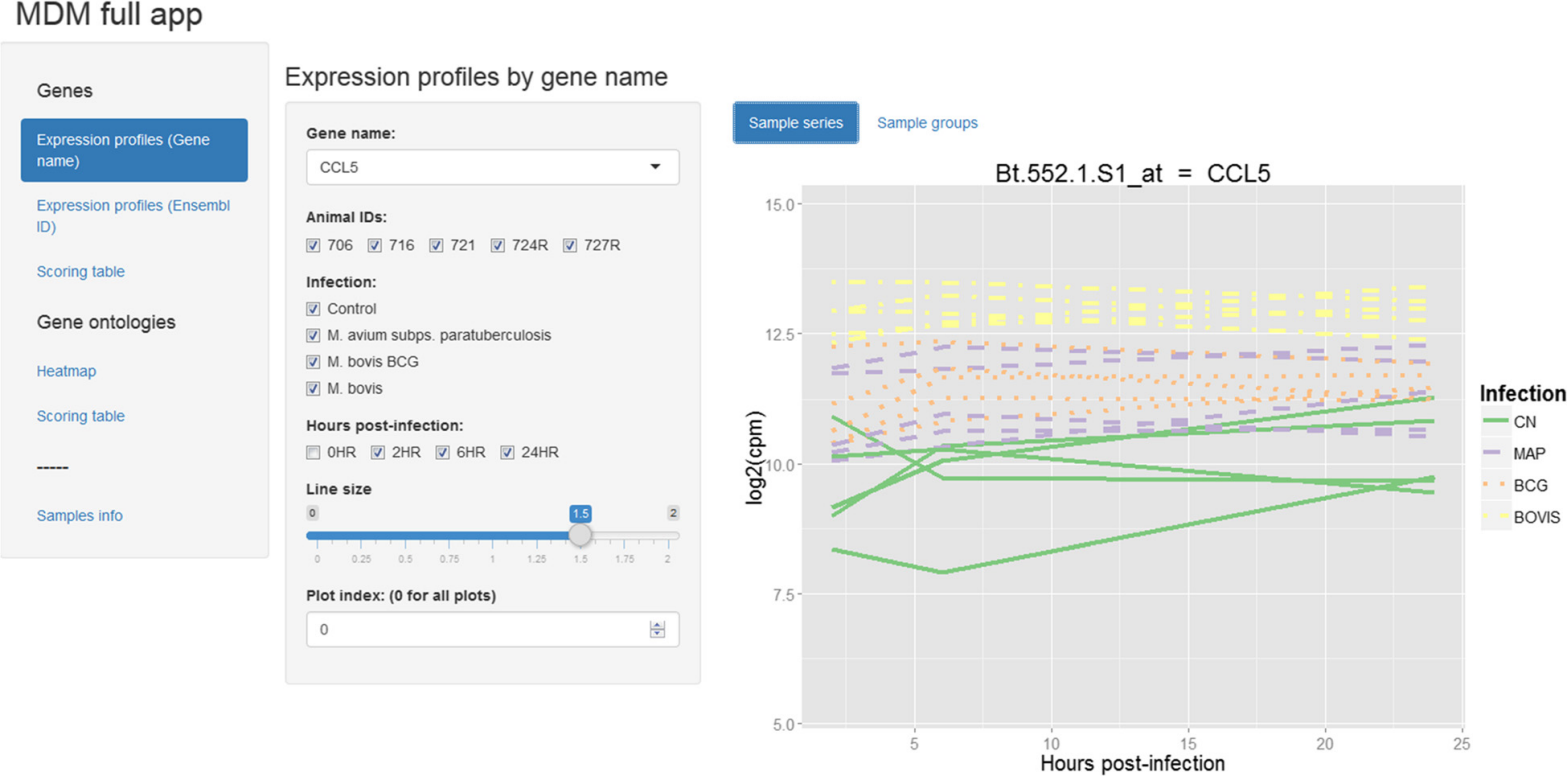
Screenshot of a sample R/Shiny application built on GOexpress results. Users may run the web application from GitHub (https://github.com/kevinrue/shiny-MDM) as shown in the main text, or from the ZIP archive provided in Additional file 4


Finally, the objects and functions available in GOexpress may be readily integrated within R/Shiny applications (http://shiny.rstudio.com), offering a dynamic interface to rapidly access the various plots and tables with minimal additional programming (Fig. 5). Shiny applications may be distributed as ZIP archives or hosted on web servers, providing a flexible interface for collaboration and exchange of experimental data and results (Additional file 4).

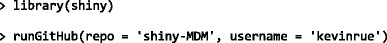


## Discussion

The widespread adoption of microarray and more recently RNA-seq for gene expression analysis has witnessed parallel development of a large ecosystem of methodologies and software packages, all designed to extract biological knowledge from increasingly complex experimental data. Traditional GSEA methods simply use two gene lists: a target set and a background set, such that the background set is used to detect over-representation of molecular pathways in the target set (e.g., GOrilla [40], GOstats [41], GOseq [42]). More recently, integration of information from differential expression and differential splicing has been proposed to weight genes (e.g., seqGSEA [43]). However, these tools are generally limited to the analysis of a single list of target genes identified from differential expression analysis in a two-group comparison, or alternatively require summarisation to merge multiple gene lists. Time-series analyses can address this limitation through characterisation of correlated differential gene expression profiles, which can be used for GO term enrichment (e.g., STEM [44]). Although the STEM program represents a powerful approach for investigating functional enrichment in groups of co-regulated genes across continuous experimental factors, it uses GO annotations largely as a descriptive summarisation of gene groups.

In contrast to the GSEA tools described above, GOexpress does not estimate or evaluate enrichment in gene lists; instead, it uses GO annotations in a functional class scoring (FCS) approach to identify terms containing genes that best classify multiple groups of samples according to any type of experimental factor. A comparison of GOexpress features with existing GO analysis software is provided in Table 2.

**Table 2:** Comparative table detailing features of different GO analysis software tools

Software	Multiple organisms	Custom annotations	Platform	Statistical method	Visualisation	Flexible threshold	Multi-level factors	Environment	Application
GOexpress (2015)	Yes	Yes	Microarray RNA-seq	Gene permutation; RF/One-way ANOVA	Gene expression; GO	Yes	Yes	R/Bioconduct r Web-app (R/Shiny)	Ranking and visualisation of genes and GO termswith expression levels that best classify multiple experimental groups
MLseq (2014)	No	No	RNA-seq	Choose from one of several algorithms (SVM, bagSVM, RF, CART)	No	No	Yes	R/Bioconductor	Application of several ML methods to RNA-seq data (using a read count table)
seqGSEA (2014)	Yes	Yes	RNA-seq	Subject permutation; Use a statistic based on the negative binomial distribution to find differentially spliced genes between two groups	Gene ranking; Gene set ranking	No	No	R/Bioconductor	Gene set enrichment analysis of high-throughput RNA-seq data by integrating differential expression and splicing
GOseq (2010)	Yes	Yes	RNA-seq	Probability weighting function (PWF); Resampling; Wallenius distribution or random sampling to choose a null distribution to find under and over representation of GO categories	No	No	No	R/Bioconductor	Detection of GO and/or other user defined categories which are over/under represented in RNA-seq data
GOrilla (2009)	Yes	No	Microarray RNA-seq	Exact mHG *P*-value computation	GO (enrichment)	Yes	No	Web-based	Identification and visualisation of enriched GO terms in ranked lists of genes
GOstats (2007)	Yes	Yes	Microarray	Hypergeometric test	Gene ontology (enrichment)	Yes	No	R/Bioconductor	Tools for interacting with GO and microarray data. A variety of basic manipulation tools for graphs, hypothesis testing and other simple calculations
STEM (2006)	Yes	Yes	Microarray	STEM clustering (assignment to predefined set of model profiles); *k*-means clustering	Gene expression cluster visualisation; integration with GO (enrichment)	Yes	No	Java	Clustering, comparison, and visualisation of short time series gene expression data from microarray experiments (~8 time points or fewer)
GSA (2007)	No	Yes	Microarray	Maxmean	GO (enrichment)	Yes	Yes	R/CRAN	Identification of gene sets where most genes or either positively or negatively correlate in a coordinated manner with higher values of phenotype.

*Abbreviations: RF* random forest, *ANOVA* analysis of variance, *SVM* support vector machines, *bagSVM* bagging support vector machines, *CART* classification and regression trees

It is well established that methodologies based on supervised learning of expression data are useful techniques for identification of biologically-relevant markers to differentiate and predict class membership in multi-level classification [45–47]. Furthermore, the RF algorithm has been shown to be one of the most robust multi-classifier algorithms for the identification of class predictors [48]. The use of supervised learning approaches such as RF for feature selection is particularly relevant for biological studies where group membership is defined by the experimental design and the number of observation is much smaller than the number of candidate predictors [49]. Notably, GOexpress is demonstrated here using an experimental design that consists of five biological replicates per experimental group, with gene expression measurements from 11,842 microarray probesets. Although there is debate concerning the optimal sample size for RF [49], we would recommend sample sizes of at least five biological replicates to accurately estimate out-of-bag (OOB) classification error rates.

In GOexpress, each GO term is individually scored by the average capacity of genes associated with the term to classify the predefined groups of samples. Consequently, if a particular GO term is associated with a number of genes that emerge among the best ranked predictors of class membership, this GO term will also be present among the top-ranking GO categories, indicative of robust differences in the corresponding cellular functions or molecular pathways (Fig. 2). Unfortunately, FCS approaches are dependent on the underlying properties of the annotations (e.g., pathway size); therefore, requiring users to choose from a complex range of univariate and multivariate pathway-level statistics [1]. GOexpress, therefore, also allows users to provide their own scoring function, as an alternative to the default averaging of feature scores. It is also important to note that the GO initiative is a rapidly developing resource, which still contains many entries that are only inferred from electronic annotation (IEA) for many species, as opposed to experimentally-validated annotations (inferred from direct assay, IDA). Therefore, careful use of appropriate gene annotations is critical for reliable results [5]. An additional feature of GOexpress is the probability of GO term scoring and ranking that may be assessed by estimation of permutation-based *P*-values. Although more computationally intensive than the use of pre-computed statistical distributions such as the minimum hypergeometric (mHG) statistical framework used by GOrilla, this assumption-free approach enables support of any set of annotations for which the underlying statistical distribution is unknown.

To the best of our knowledge, no currently available software package provides similar integration of multi-level sample classification directly based on gene expression data from both microarray and RNA-seq experiments (with support for new platforms through user-provided custom annotations). Importantly, the data-driven visualisation functions provided in the GOexpress package do not transform the input expression data, assuming this task was performed using dedicated tools such as edgeR [12], limma [13], Cufflinks [50], or DEseq2 [51]. GOexpress, therefore, can be seamlessly integrated within existing computational biology pipelines, and can be used for development of dynamic Shiny web-applications that may be distributed online and offline, promoting collaboration and accessibility of high-throughput biological data and results within and between research groups.

## Conclusion

We have introduced GOexpress, an R/Bioconductor package for identification and visualisation of gene expression profiles that best classify sample groups according to any known experimental factor. In contrast to most GO term summarisation approaches, GOexpress integrates prior biological knowledge and gene expression data from individual sample replicates to rank molecular pathways based on the capacity of functionally-related groups of genes to classify multiple sample groups. Notably, the use of multiple genes for GO-based classification improves the robustness and biological relevance of the resulting interpretations and predictions.

## Availability and requirements

- Project name: GOexpress
- Project home page: http://bioconductor.org/packages/release/bioc/html/GOexpress.html
- Operating system(s): Platform independent
- Programming language: R
- Other requirements: R 3.1 or higher, Bioconductor 3.0 or higher
- License: GPL (> = 3)
- Any restrictions to use by non-academics: None

### Ethical approval for animal work

All animal procedures were carried out according to the provisions of the Irish Cruelty to Animals Act (Department of Health and Children licence number B100/3939) and ethical approval for the study was obtained from the UCD Animal Ethics Committee (protocol number AREC-P-07-25-MacHugh).

## Additional files

Additional file 1: Script used to perform the analysis shown in the paper. The script includes preparation of local gene and GO annotations, the main analysis, the computation of permutation-based *P*-values, the filtering of GO terms, and the various visualisation functions at both gene and GO levels. (R 4 kb) Additional file 2: Package source code for GOexpress release 1.2.1. (GZ 2281 kb) Additional file 3: Pseudocode calculating the rank of GO terms and average score from the rank of gene features, shown in Fig. 2 and Additional file 7. (DOCX 15 kb) Additional file 4: Compressed ZIP archive containing a sample Shiny application built on the data demonstrated in the paper. The archive also includes serialised R objects saved to files, such as the ExpressionSet described in the paper. (ZIP 7908 kb) Additional file 5: Scoring table for gene features produced by GOexpress using the ExpressionSet described in the paper (see Additional file 1). (XLSX 622 kb) Additional file 6: Comparison of the importance score from the random forest algorithm (i.e., the mean decrease in Gini index) to the *F*-ratio from a one-way ANOVA. Probesets with large importance score (i.e., good classifiers) generally show a high *F*-ratio, indicative of high variance among the means of each group compared to the variance within the samples. (PDF 1215 kb) Additional file 7: Scoring table for GO terms produced by GOexpress using the ExpressionSet described in the paper (see Additional file 1). (XLSX 871 kb) Additional file 8: Scoring table comparing the results of the GSA package to those of GOexpress. A short description of the column headers in the ‘GSA positive results’ worksheet is provided in the ‘legend’ worksheet. (XLSX 78 kb)

## Abbreviations

CPM: counts per million
FCS: functional class scoring
GSEA: gene set enrichment analysis
GO: gene ontology
MDM: monocyte-derived macrophage
RF: random forest
RNA-seq: RNA-sequencing
