# Supplementary material for: GOexpress: an R/Bioconductor package for the identification and visualisation of robust gene ontology signatures through supervised learning of gene expression data
Source: BMC Bioinformatics. 2016 Mar 11;17:126. doi: 10.1186/s12859-016-0971-3 (PMC4788925; doi:10.1186/s12859-016-0971-3)
Supplement: Additional file 2: — Package source code for GOexpress release 1.2.1. (GZ 2281 kb) [file 12859_2016_971_MOESM2_ESM.gz › GOexpress/inst/doc/GOexpress-UsersGuide.pdf]

GOexpress: identify and visualise robust gene ontology signatures through supervised clustering of gene expression data

Kévin Rue-Albrecht, Paul A. McGettigan, Belinda Hernández,
David A. Magee, Nicolas C. Nalpas, Andrew C. Parnell,
Stephen V. Gordon, and David E. MacHugh

April 19, 2015

Contents

1 Introduction 2
1.1 The origin and purpose of *GOexpress* . . . 2
1.2 Purpose of this document . . . 2

2 Before you start 3
2.1 Installation . . . 3
2.2 Getting help . . . 3
2.3 Citing *GOexpress* . . . 3

3 Quick start 4
3.1 Input data . . . 4
3.2 Main analysis . . . 5
3.2.1 Preparing the grouping factor to analyse . . . 5
3.2.2 Running the random forest algorithm using local annotations . . . 5
3.2.3 Important notes in the absence of local annotations . . . 6
3.3 Permutation-based P-value for ontologies . . . 7
3.4 Filtering of results . . . 7
3.4.1 Filtering of the result object . . . 7
3.4.2 Quick filtering of the ontology scoring table . . . 8
3.5 Details of the top-ranking GO terms . . . 8
3.6 Hierarchical clustering of samples based on gene expression associated with a GO term . . . 9
3.7 Details of genes associated with a GO term . . . 11
3.8 Expression profile of a gene by sample group . . . 12
3.8.1 Using the unique feature identifier . . . 12
3.8.2 Using the associated gene name . . . 14
3.9 Expression profile of a gene by individual sample series . . . 15
3.9.1 Using the unique feature identifier . . . 15
3.9.2 Using the associated gene name . . . 16
3.10 Comparison of univariate effects on gene expression . . . 16

4 Additional controls and advanced functions 17
4.1 Custom annotations . . . 17
4.1.1 Generating custom annotations . . . 18
4.1.2 Using custom annotations . . . 18
4.2 Using subsets of samples . . . 19
4.3 Overlapping genes between GO terms . . . 19
4.4 Distribution of scores . . . 20
4.5 Reordering scoring tables . . . 21
4.5.1 Reordering by score . . . 21
4.5.2 Reordering by P-value . . . 21

4.5.3 Reordering and breaking ties . . . 22
4.6 Subsetting an ExpressionSet to specific sample groups . . . 22

5 Statistics 22
5.1 Overview . . . 22
5.2 Data-driven visualisation functions . . . 22
5.3 Random Forest . . . 23
5.4 One-way Analysis of Variance (ANOVA) . . . 23

6 Integration with other packages 23
6.1 shiny . . . 23

7 Notes 23
7.1 Authors' contributions . . . 23
7.2 Acknowledgement . . . 24
7.3 Session information . . . 24

1 Introduction

1.1 The origin and purpose of GOexpress

The idea leading to the *GOexpress* *R* package emerged from a set of plotting functions I regularly copy-pasted across various complex multifactorial transcriptomics studies from both microarray and RNA-seq platforms. Those functions were repeatedly used to visualise the expression profile of genes across groups of samples, to annotate technical gene identifiers from both microarray and RNA-seq platforms (i.e. probesets, Ensembl gene identifiers) with their associated gene name, and to evaluate the clustering of samples based on genes participating in a common cellular function or location (i.e. gene ontology). While developing the *GOexpress* package and discussing its features with colleagues and potential users, a few more features were added, to enhance and complement the initial functions, leading to the present version of the package.

Complex multifactorial experiments have become the norm in many research fields, thanks to the decrease in cost of high-throughput transcriptomics platforms and the barcoding/multiplexing of samples on the RNA-seq platform. While much effort has been (correctly!) spent on the development of adequate statistical frameworks for the processing of raw expression data, much of the genewise exploration and visualisation is left to the end-user. However, data summarisation and visualisation can be a daunting task in multifactorial experiments, or require large amounts of copy-pasting to investigate the expression profile of a handful or genes and cellular pathways.

Developed and tested on multiple RNA-seq and microarray datasets, *GOexpress* offers an extendable set of data-driven plotting functions readily applicable to the output of widely used analytic packages estimating (differential) gene expression. Once the initial analysis and filtering of *GOexpress* results is complete — literaly two command lines —, each gene and gene ontology is accessible by a single line of code to produce high-quality graphics and summary tables. In short, *GOexpress* is a software package developed based on real experimental datasets to ease the visualisation and interpretation of multifactorial transcriptomics data by bioinformaticians and biologists, while striving to keep it a simple, fast, and intuitive toolkit.

Notably, the use of the *biomaRt* package enables *GOexpress* to support and annotate gene expression identifiers from any species and any microarray platform present in the Ensembl BioMart server (`http://www.ensembl.org/biomart/martview`), while custom annotations may also be provided for the analysis of species or platforms not supported yet, the clustering of non-transcriptomics datasets (e.g. proteomics), or the comparison of panels of biomarkers independent from gene ontology annotations.

1.2 Purpose of this document

This User's Guide was intended as a helpful description of the main features implemented in the *GOexpress* package, as well as a tutorial taking the user through a typical analysis pipeline that *GOexpress* was designed for. While an example usage will be provided for each function of the package, the many arguments of each function cannot

realistically be demonstrated in this Guide, and we kindly ask users to also read the individual help pages accompanying the corresponding package functions for further details.

2 Before you start

2.1 Installation

Installing *GOexpress* should be as easy as running the two lines below:

```
> source("http://bioconductor.org/biocLite.R")
> biocLite("GOexpress")
```

Installation issues should be reported to the *Bioconductor* mailing list.

2.2 Getting help

The *GOexpress* package is still relatively young and may require some fine-tuning or bug fixes. Please contact the maintainer with a copy of the error message and the command run.

```
> maintainer("GOexpress")
[1] "Kevin Rue-Albrecht <kevin.rue@ucdconnect.ie>"
```

Despite our efforts to repeatedly test the package on in-house datasets of both microarray and RNA-seq platform, and of human and bovine origin, many of the model species and gene expression platforms have not been tested yet. We welcome feedback!

Interesting suggestions for additional package functions, or improvement of existing ones are most welcome and may be implemented when time allows. Alternatively, we also encourage users to fork the GitHub repository of the project, develop and test their own feature(s), and finally generate a pull request to integrate it to the original repository (https://github.com/kevinrue/GOexpress).

As for all *Bioconductor* packages, the *Bioconductor* support site is the best place to seek advice with a large and active community of *Bioconductor* users. More detailed information is available at:

http://www.bioconductor.org/help/support.

2.3 Citing GOexpress

The work underlying *GOexpress* has not been formally published yet. A manuscript has been submitted for peer-review. In the meantime, users of the *GOexpress* package are encouraged to cite it using the output of the `citation` function in the *utils* package, as follows:

```
To cite package ‘GOexpress’ in publications use:

  Kevin Rue-Albrecht (2014). GOexpress: Visualise microarray and RNAseq data
  using gene ontology annotations. R package version 1.2.1.
  https://github.com/kevinrue/GOexpress

A BibTeX entry for LaTeX users is

  @Manual{,
    title = {GOexpress: Visualise microarray and RNAseq data using gene ontology annotations},
    author = {Kevin Rue-Albrecht},
    year = {2014},
    note = {R package version 1.2.1},
    url = {https://github.com/kevinrue/GOexpress},
  }
```

3 Quick start

3.1 Input data

Despite their different underlying technologies, microarray and RNA-seq analytic pipelines typically yield a matrix measuring the expression level of many gene features in each sample. Commonly, this expression matrix is filtered to retain only genes qualified as "informative" (e.g. > 1 cpm in at least N replicates; N being the number of replicates for a given set of experimental conditions); and genes lowly expressed are removed to limit the False Discovery Rate (FDR) of differentially expressed genes induced by the larger variability of expression at the lower end of the dynamic range.

GOexpress requires this filtered normalised expression matrix to be accompanied by an *AnnotatedDataFrame* object of the *Biobase* package providing phenotypic information for each of those samples (e.g. unique identifier, treatment, time-point). *GOexpress* expects those two variables in an *ExpressionSet* container of the *Biobase* package, both simplifying the manipulation of the data and, most importantly, ensuring interoperatibility with other packages that handle *Bioconductor ExpressionSet* objects. The other fields of the *ExpressionSet* container may be left empty as *GOexpress* does not currently access them. Instructions to create *AnnotatedDataFrame* and *ExpressionSet* objects are detailed in the vignettes of the *Biobase* package.

To use the analytical part of the *GOexpress* package, the phenotypic data-frame — `phenodata` slot of the *ExpressionSet* — must contain at least one column containing an experimental factor made of two or more levels in the strict meaning of "factor" and "levels" in the *R* programming language. The above *ExpressionSet* and the name of the column containing such a factor are the minimal two input variables required for the `GO_analyse` function to work. Additional arguments may be required, in particular for microarray datasets, but those are discussed in section 3.2.3.

In the examples below, we will use the toy dataset `AlvMac` provided with the package and made of a subset of 100 bovine Ensembl gene identifiers (rows) measured in 117 samples (columns), extracted from a larger RNA-seq experiment (see help page for the `AlvMac` object). This toy *ExpressionSet* also includes an *AnnotationDataFrame* detailing a number of phenotypic information fields describing each sample.

Let us load the *GOexpress* package and import the toy dataset in the workspace:

```
> library(GOexpress) # load the GOexpress package
> data(AlvMac) # import the training dataset
```

Now, the expression matrix and phenotypic data of the *ExpressionSet* container can be accessed using dedicated functions from the *Biobase* package:

```
> exprs(AlvMac)[1:5,1:5] # Subset of the expression data

                   N1178_CN_24H N1178_CN_2H N1178_CN_48H N1178_CN_6H N1178_MB_24H
ENSBTAG00000027610     6.756882    6.622802     6.563849    6.542920     7.340347
ENSBTAG00000019253     3.679282    3.395152     3.296391    3.595889     3.880105
ENSBTAG00000025564     7.125069    7.440622     7.302704    7.337605     7.925776
ENSBTAG00000047107     5.405097    7.583981     4.772563    6.433678     8.222054
ENSBTAG00000016683     8.753575   10.226074     8.130427    9.325824     9.332076
```

```
> head(pData(AlvMac)) # Subset of the phenotypic information

                     File       Sample Animal Treatment Time  Group Timepoint
N1178_CN_24H N1178_CN_24H N1178_CN_24H  N1178        CN  24H CN_24H        24
N1178_CN_2H   N1178_CN_2H  N1178_CN_2H  N1178        CN   2H  CN_2H         2
N1178_CN_48H N1178_CN_48H N1178_CN_48H  N1178        CN  48H CN_48H        48
N1178_CN_6H   N1178_CN_6H  N1178_CN_6H  N1178        CN   6H  CN_6H         6
N1178_MB_24H N1178_MB_24H N1178_MB_24H  N1178        MB  24H MB_24H        24
N1178_MB_2H   N1178_MB_2H  N1178_MB_2H  N1178        MB   2H  MB_2H         2
```

An advantage of the *ExpressionSet* container is that it takes care of the compatibility between the expression matrix and the phenotypic information data-frame. For instance, it will check that samples names do not differ between expression matrix and phenotypic information.

Users can visually inspect that adequate row names are used in the expression matrix:

```
> head(rownames(exprs(AlvMac))) # Subset of gene identifiers
```

```
[1] "ENSBTAG00000027610" "ENSBTAG00000019253" "ENSBTAG00000025564" "ENSBTAG00000047107"
[5] "ENSBTAG00000016683" "ENSBTAG00000016085"
```

comment: In the training dataset, the `Time` column of `pData(targets)` is an R factor while the `Timepoint` column is a numeric vector. The fomer is useful for grouping the samples for the analysis, while the latter is better suited to plot gene expression profiles respecting the relative distance between the time-points. See section 3.8.1 for examples using of a numeric value or a factor as the variable of the X-axis.

3.2 Main analysis

3.2.1 Preparing the grouping factor to analyse

In this example, we search for GO terms enriched in genes that cluster best the samples according their `Treatment` level. But first, let us make sure that the `Treatment` column of `pData(targets)` is indeed an *R* factor:

```
> is.factor(AlvMac$Treatment) # assertion test
```

```
[1] TRUE
```

```
> AlvMac$Treatment # visual inspection
```

```
  [1] CN CN CN CN MB MB MB MB TB TB TB TB CN CN CN CN MB MB MB MB TB TB TB TB CN CN CN CN
 [29] MB MB MB MB TB TB TB TB CN CN CN CN MB MB MB MB TB TB TB TB CN CN CN CN MB MB MB MB
 [57] TB TB TB TB CN CN CN CN MB MB MB MB TB TB TB TB CN CN CN MB MB MB TB TB TB CN CN CN
 [85] CN MB MB MB MB TB TB TB TB CN CN CN CN MB MB MB MB TB TB TB TB CN CN CN CN MB MB MB
[113] MB TB TB TB TB
Levels: CN MB TB
```

In this case, it is already a properly formatted factor. If that was not the case, the following line of code would convert the column to an *R* factor and allow to continue the analysis (note that in some cases, it may be preferrable to order the different levels of a factor, for an example see factor `Time`):

```
> AlvMac$Treatment <- factor(AlvMac$Treatment)
```

3.2.2 Running the random forest algorithm using local annotations

Now, we use the random forest statistical framework to score each gene feature on its ability to cluster samples from different treatments separately, before summarising this information at the ontology level. The ensuing analysis therefore considers the `Treatment` factor, irrespective of the `Time` and `Animal` factors (we search for time- and animal-independent discriminants of infection). Alternatively, a subset of samples from the input *ExpressionSet* may be specified to address more specific hypotheses. In this example, we use locally saved copies of gene and gene ontology annotations previously downloaded from Ensembl annotation release 75 using the *biomaRt* package (See section 4.1 to generate suitable local annotations, and the benefits of using them).

```
> set.seed(4543) # set random seed for reproducibility
> AlvMac_results <- GO_analyse(
+     eSet = AlvMac, f = "Treatment",
+     GO_genes=AlvMac_GOgenes, all_GO=AlvMac_allGO, all_genes=AlvMac_allgenes)
```

```
Using custom GO_genes mapping ...
91 features from ExpressionSet found in the mapping table.
Using custom GO terms description ...
Analysis using method randomForest on factor Treatment for 100
genes. This may take a few minutes ...
ntree      OOB      1      2      3
  100:  57.26% 28.21% 61.54% 82.05%
  200:  52.99% 25.64% 53.85% 79.49%
  300:  52.14% 25.64% 56.41% 74.36%
  400:  53.85% 25.64% 58.97% 76.92%
  500:  52.99% 25.64% 56.41% 76.92%
  600:  53.85% 28.21% 56.41% 76.92%
```

```
  700:  54.70% 28.21% 58.97% 76.92%
  800:  53.85% 25.64% 58.97% 76.92%
  900:  52.99% 25.64% 58.97% 74.36%
 1000:  53.85% 25.64% 61.54% 74.36%
Using custom gene descriptions ...
Merging score into result table ...
```

At this stage, it is a good idea to save the result variable into an *R* data-file using the save function. Mostly because the stochastic aspect of the sampling approach implemented by the *randomForest* package may return slightly different scores in each run (as opposed to the use of ANOVA F-score).

The output variable of the analysis summarises the parameters of the analysis and can easily be browsed with standard *R* functions:

```
> names(AlvMac_results) # Data slot names

 [1] "GO"       "mapping" "genes"   "factor"  "method"  "subset"  "rank.by" "FUN.GO"
 [9] "ntree"    "mtry"

> head(AlvMac_results$GO[, c(1:5, 7)], n=5) # Ranked table of GO terms (subset)

           go_id ave_rank ave_score total_count data_count     namespace_1003
1308  GO:0004113      2.0  3.461982           1          1 molecular_function
3498  GO:0009214      2.0  3.461982           1          1 biological_process
6341  GO:0032682      7.0  1.264116           1          1 biological_process
11129 GO:0070427      7.5  1.686214           2          2 biological_process
6532  GO:0033091     11.0  1.001124           1          1 biological_process

> head(AlvMac_results$genes[, c(1:3)], n=5) # Ranked table of genes (subset)

                      Score Rank external_gene_name
ENSBTAG00000007239 7.490250    1              TSG-6
ENSBTAG00000025762 3.461982    2                CNP
ENSBTAG00000047107 3.362813    3              TNIP3
ENSBTAG00000016683 2.371304    4              BIKBA
ENSBTAG00000019872 2.329323    5            PIK3AP1

> head(AlvMac_results$mapping) # Gene to gene ontology mapping table (subset)

             gene_id      go_id
1 ENSBTAG00000020495 GO:0005515
2 ENSBTAG00000020495 GO:0006661
3 ENSBTAG00000020495 GO:1900027
4 ENSBTAG00000020495 GO:0032587
5 ENSBTAG00000020495 GO:0019902
6 ENSBTAG00000020495 GO:0035091
```

comment: In the tables of GO terms and genes above, the column containing the name of the GO terms and the column containing the description of the genes are hidden, as their content is very long in some cases, affecting the readability of this document.

3.2.3 Important notes in the absence of local annotations

If no annotations mapping gene features identifiers to gene ontology identifiers are provided, GO_analyse will connect to the Ensembl server to fetch appropriate annotations in a semi-automated procedure (See arguments dataset and microarray of the GO_analyse function). Typically, the first feature identifier in the ExpressionSet is used to determine the corresponding species and type of data. This is a fairly straightforward process for Ensembl gene identifiers (e.g. in the prefix 'ENSBTAG', 'BT' indicates *Bos taurus*). Hence, the simplest use of the GO_analyse is:

```
> AlvMac_results <- GO_analyse(eSet = AlvMac, f = "Treatment")
```

warning: Without local annotations, connecting to the Ensembl server and downloading the annotations significantly impacts the runtime of the function.

However, it can be more difficult to identify the microarray used to obtain a certain dataset, as many different Affymetrix chips contain probesets named with the pattern "AFFX.*" for instance. In cases where the microarray platform cannot be detected automatically, we recommend using the `microarray` argument of the `GO_analyse` function. The list of valid values for the microarray argument is available in the `microarray2dataset` data frame which can be loaded in the workspace using:

```
> data(microarray2dataset)
```

3.3 Permutation-based P-value for ontologies

To assess the significance of GO term ranking — or scoring —, we implemented a permutation-based function randomising the gene feature ranking, and counting how many times each GO term is ranked (scored) equal or higher than the real rank (score).

Critically, the function should be applied directly to the output of the `GO_analyse` function prior to filtering, in order to use the full list of gene features as a background for permutation:

```
> AlvMac_results.pVal = pValue_GO(result=AlvMac_results, N=100)
```

warning: The `pValue_GO` function is relatively lengthy. However, it is suggested to calculate P-values on the basis of at least 1,000 permutation (approximately 50 min on a standard Ubuntu server) to obtain reach minimal non-zero P-values as low as 0.001.

Given that many genes are associated to various gene ontologies due to the hierarchical relationships in the three Direct Acyclic Graphs (DAGs), a permutation-based approach appears the best suited strategy to assess the significance of ontology-related genes to display a specific average rank (or score). As further discussed in the next section, this approach also addresses the issue of the considerable range of gene counts associated with each known gene ontology.

3.4 Filtering of results

3.4.1 Filtering of the result object

The `subset_scores` function allows users to filter for GO terms passing certain criteria (e.g. maximal P-value, minimal gene counts, type of ontology). A `filters.GO` slot will be created in the filtered result object, stating the filters and cutoff values applied. A filtered object may be further filtered, and the `filters.GO` slot will be updated accordingly.

Importantly, an early-identified bias of the scoring function is that GO terms associated with fewer genes are favored at the top of the ranking table. This is due to the fact that it is much easier for a group of 5 genes (e.g. "`B cell apoptotic process`") to have an high average rank — and average score — than it is for a group of 6,000 genes (e.g. "`protein binding`"). Indeed, the highest possible average rank of 5 genes is 3 while it is 3,000 for a group of 6,000 genes. The calculation of P-values using the `pValue_GO` partially controls that bias, as smaller groups of ontology-related genes may appear by chance at a higher average rank than observed using the ranking based on actual gene expression.

Furthermore, in our experience, this bias presents some benefits. First, it implicitely favors specific and well-defined GO terms (e.g. "`negative regulation of T cell apoptotic process`") as opposed to vague and uninformative GO terms (e.g. "`cytoplasm`"). Secondly, we observed many top-ranking GO terms associated with a single gene. Those GO terms are consequently susceptible to single-gene events and artefacts in the expression data, as opposed to GO terms with a reasonable number of associated genes. Using the above filtering function, it is straightforward to filter out those GO terms with only a handful of associated genes, in combination with a standard P-value filter:

```
> BP.5 <- subset_scores(
+     result = AlvMac_results.pVal,
+     namespace = "biological_process",
+     total = 5, # requires 5 or more associated genes
+     p.val=0.05)
> MF.10 <- subset_scores(
+     result = AlvMac_results.pVal,
+     namespace = "molecular_function",
```

```
+     total = 10,
+     p.val=0.05)
> CC.15 <- subset_scores(
+     result = AlvMac_results.pVal,
+     namespace = "cellular_component",
+     total = 15,
+     p.val=0.05)
```

Finally, the inherent hierarchical structure and "granularity" of gene ontology terms can be browsed conveniently by using increasingly large values of the `total` filter. Note that this filter retains only GO terms associated with a minimal given count of genes in the gene-GO mapping table. It is also possible to use the `data` argument to filter for GO terms associated with a certain count of genes in the given expression dataset, although this approach is obviously more data-dependent and less robust.

warning: To optimise the use of memory space, after removing all ontologies not passing the criteria, the function will also discard from the filtered result object all gene features not associated with the remaining gene ontologies, and the mapping information related to gene ontologies absent from the filtered data. However, those data will still be left in the original object containing unfiltered raw results.

3.4.2 Quick filtering of the ontology scoring table

As an alternative to the above cascade-filtering of gene ontologies and gene features result tables, users can extract and filter information from either the gene or the gene ontology scoring tables using the `subset` function.

An example of filtering the gene ontology result table for the top ontologies of the 'Biological Process' type, associated with at least 5 genes, and a P-value lower than 0.05:

```
> subset(
+     AlvMac_results.pVal$GO,
+     total_count >= 5 & p.val<0.05 & namespace_1003=='biological_process'
+     )
```

comment: Note that the above command will return a data-frame containing only the filtered GO terms, as opposed to the full result object returned by the `subset_scores` function.

3.5 Details of the top-ranking GO terms

Once the GO terms are ranked (and filtered), the top-ranking GO terms in the filtered object are those most enriched in genes with expression levels that best cluster the predefined groups of samples, based on the levels of the factor considered (`raw_results$factor`).

In this example, we list the top filtered "Biological Process" GO terms extracted above and their statistics (currently ranked by increasing average rank of their associated genes):

```
> head(BP.5$GO)

        go_id ave_rank ave_score total_count data_count p.val
18 GO:0034142 40.00000 1.3278388           8          7     0
32 GO:0034134 63.00000 0.7295710           5          3     0
34 GO:0070431 63.60000 0.6744856           5          2     0
68 GO:0071223 78.28571 0.2487055           7          2     0
70 GO:0010745 81.60000 0.4742608           5          1     0
71 GO:0010888 81.60000 0.4742608           5          1     0
                                                        name_1006
18                                   toll-like receptor 4 signaling pathway
32                                   toll-like receptor 2 signaling pathway
34 nucleotide-binding oligomerization domain containing 2 signaling pathway
68                                   cellular response to lipoteichoic acid
70      negative regulation of macrophage derived foam cell differentiation
71                                     negative regulation of lipid storage
       namespace_1003
```

```
18 biological_process
32 biological_process
34 biological_process
68 biological_process
70 biological_process
71 biological_process
```

3.6 Hierarchical clustering of samples based on gene expression associated with a GO term

In the previous section, we identified the GO terms most enriched for genes clustering samples according to their treatment. We will now generate for the top-ranked GO term (“`toll-like receptor 4 signaling pathway`”) a heatmap to visualise simulatenously the clustering of samples and the expression level of each gene in each sample:

```
> heatmap_GO(
+     go_id = "GO:0034142", result = BP.5, eSet=AlvMac, cexRow=0.4,
+     cexCol=1, cex.main=1, main.Lsplit=30)
```

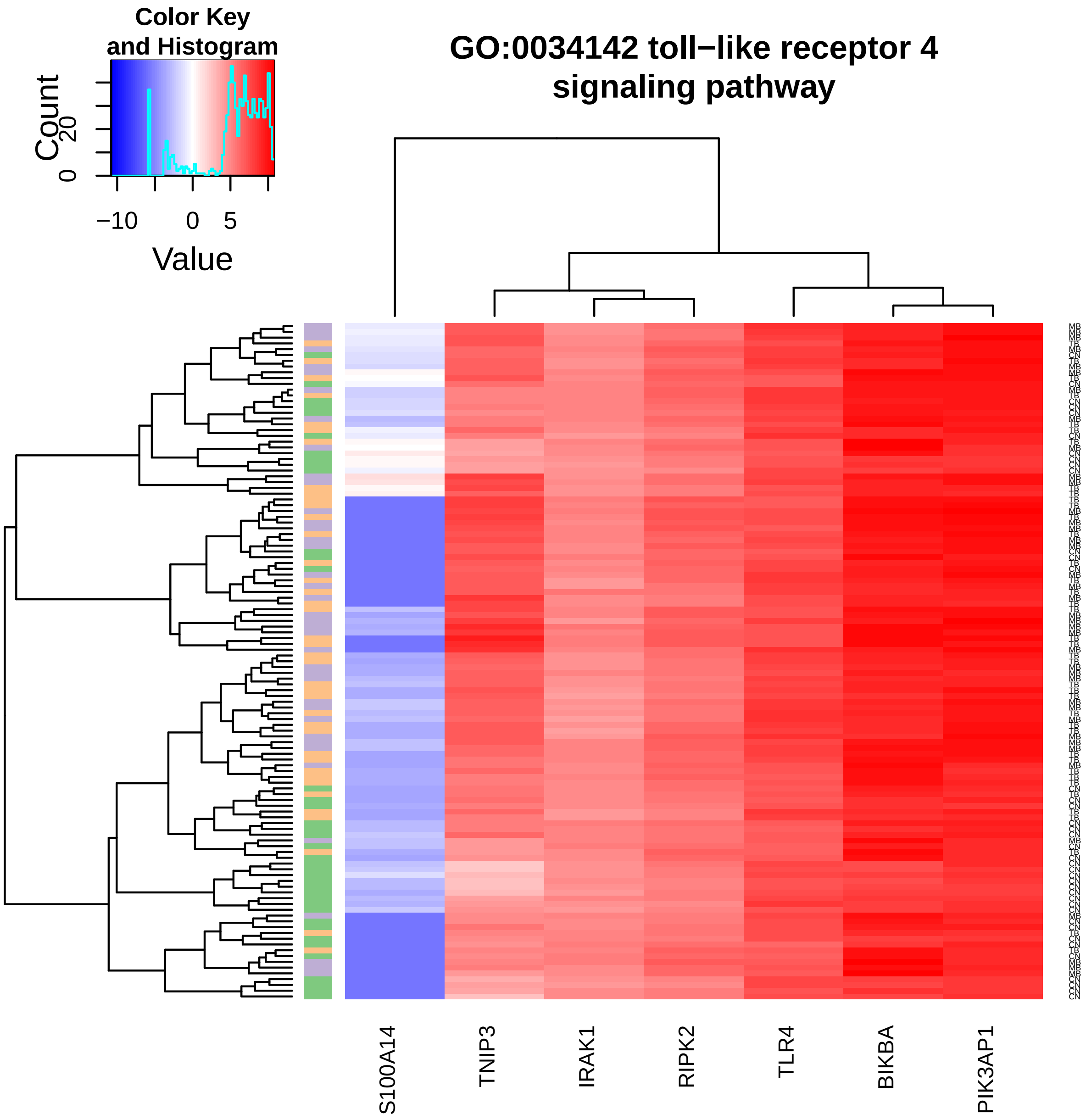

In this example, we can observe a group of "Control" (i.e. untreated; green color) samples clustering together at the bottom of the heatmap.

Re-labelling of samples by `Group` (i.e. combination of treatment and time-point) reveals that those samples are mainly 24 and 48 hours post-infection control samples:

```
> heatmap_GO(
+     go_id = "GO:0034142", result = BP.5, eSet=AlvMac, cexRow=0.4,
+     cexCol=1, cex.main=1, main.Lsplit=30,
+     labRow=AlvMac$Group)
```

Subsequently encouraging the generation of a heatmap restricted to samples from those time-points (i.e. `24H` and `48H`):

```
> heatmap_GO(
+     go_id = "GO:0034142", result = BP.5, eSet=AlvMac, cexRow=0.6,
+     cexCol=1, cex.main=1, main.Lsplit=30,
+     labRow='Group', subset=list(Time=c('24H','48H')))
```

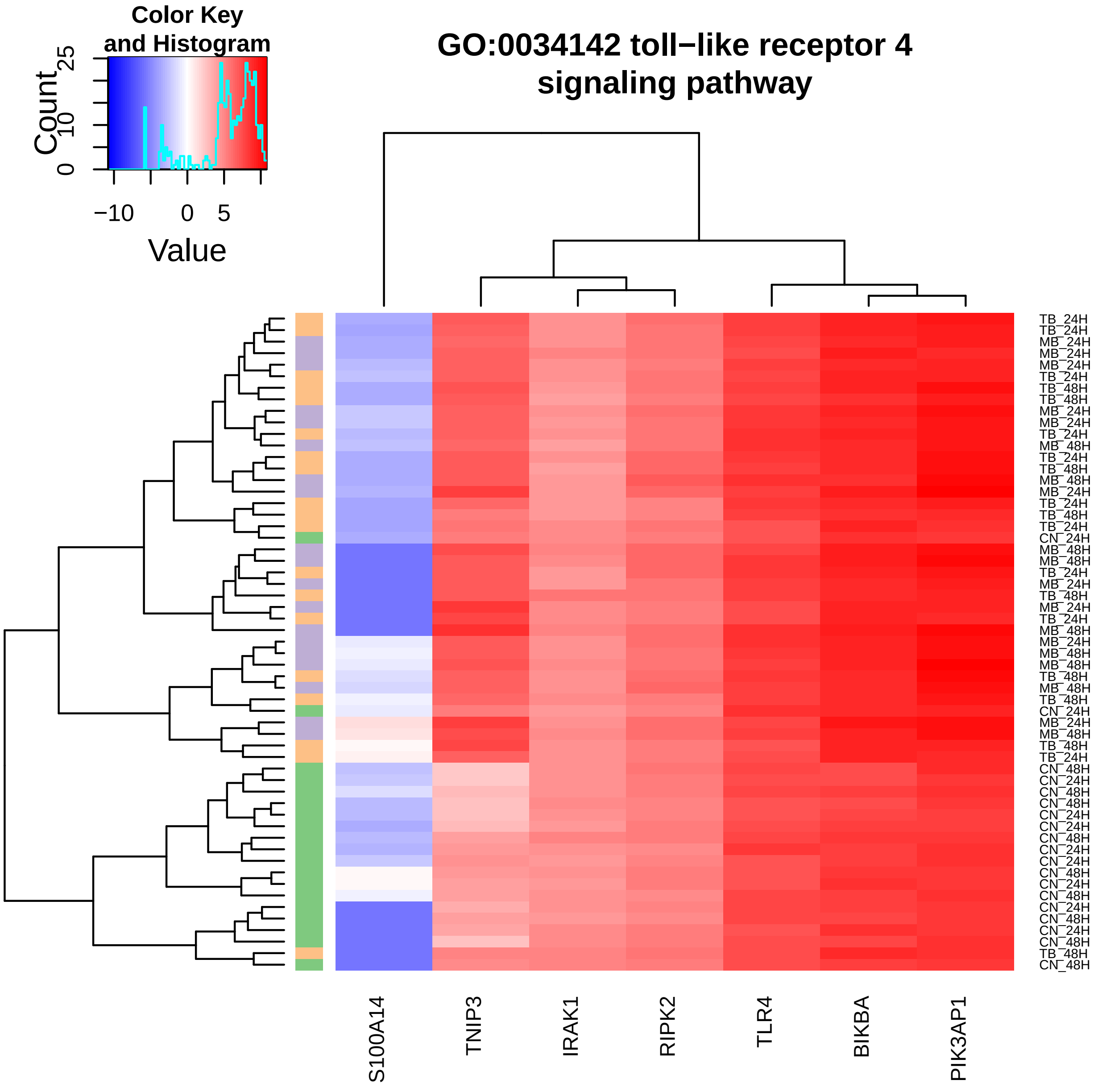

Alternatively, it is possible to focus only on the hirearchical clustering of samples. The following code will build a dendrogram clustering samples using the expression data of the subset of genes associated with the "`toll-like receptor 4 signaling pathway`" gene ontology, considering only samples obtained 24 and 48 hours post-infection, and labelling samples by `Group` rather than simply `Treatment`:

```
> cluster_GO(
+     go_id = "GO:0034142", result = BP.5, eSet=AlvMac,
+     cex.main=1, cex=0.6, main.Lsplit=30,
+     subset=list(Time=c("24H", "48H")), f="Group")
```

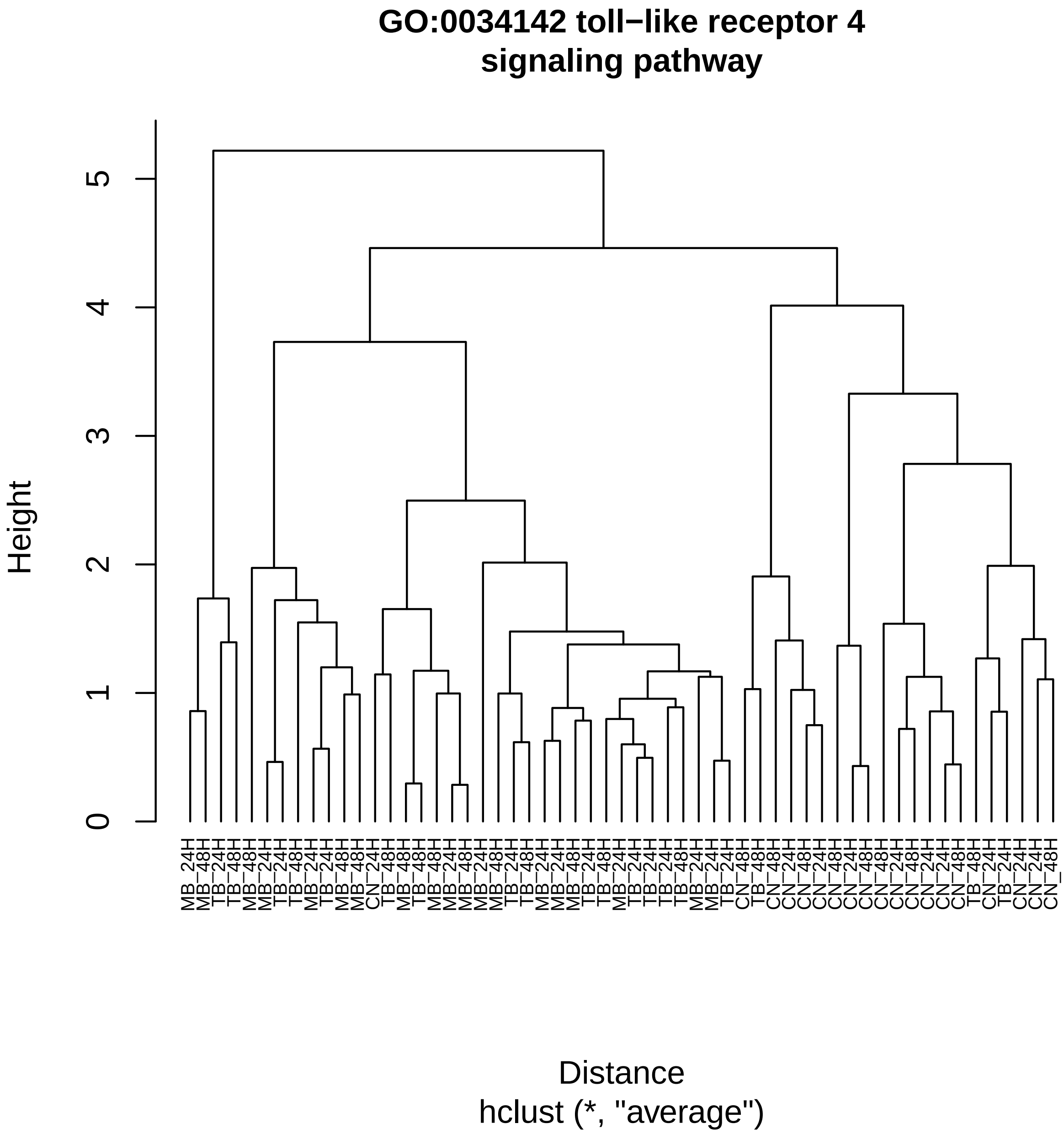

Note that labelling samples by another factor does not affect the clustering process itself, as the underlying expression data has not changed.

3.7 Details of genes associated with a GO term

Following the identification of relevant GO terms in the above sections, users may want to have a closer look at the individual genes associated with a given GO term. The default behaviour of the function is to order the gene features

by increasing rank (equivalent to decreasing score):

```
> table_genes(go_id = "GO:0034142", result = BP.5)[,c(1:3)]
```

```
                       Score Rank external_gene_name
ENSBTAG00000047107 3.3628134    3              TNIP3
ENSBTAG00000016683 2.3713040    4              BIKBA
ENSBTAG00000019872 2.3293233    5            PIK3AP1
ENSBTAG00000015271 1.0011242   11              RIPK2
ENSBTAG00000006240 0.7398141   32               TLR4
ENSBTAG00000021377 0.5009235   67            S100A14
ENSBTAG00000016085 0.3174076   97              IRAK1
ENSBTAG00000024340        NA   NA               <NA>
```

comment: In the table above, the column containing the description of the genes is hidden, as its content was very long in some cases, affecting the readability of this document.

Note that the default behaviour of the above function is to return a table of all the genes associated with the GO term based on the annotations collected. For obvious reasons, genes present in the annotations but absent from the expression dataset will be absent from the score table and consequently lack data in this result table. It is possible to restrict the above table to only genes present in the expression dataset using the `data.only` argument set to `TRUE`.

If only the feature identifiers associated with a given GO identifier are needed, users may use the function below:

```
> list_genes(go_id = "GO:0034142", result = BP.5)
```

```
[1] "ENSBTAG00000047107" "ENSBTAG00000016683" "ENSBTAG00000016085" "ENSBTAG00000021377"
[5] "ENSBTAG00000019872" "ENSBTAG00000006240" "ENSBTAG00000015271"
```

3.8 Expression profile of a gene by sample group

3.8.1 Using the unique feature identifier

In the above section, we listed the genes associated with a particular gene ontology. In our example, the respective score and rank of each gene estimates the capacity of the gene to cluster the samples according to the treatment factor. The genes that best cluster the samples will have the highest scores and the lowest ranks. Those genes will likely produce the expression profiles with the most consistent differential expression between the treatment groups over time. Here is one example:

```
> expression_plot(
+     gene_id = "ENSBTAG00000047107", result = BP.5, eSet=AlvMac,
+     x_var = "Timepoint", title.size=1.5,
+     legend.title.size=10, legend.text.size=10, legend.key.size=15)
```

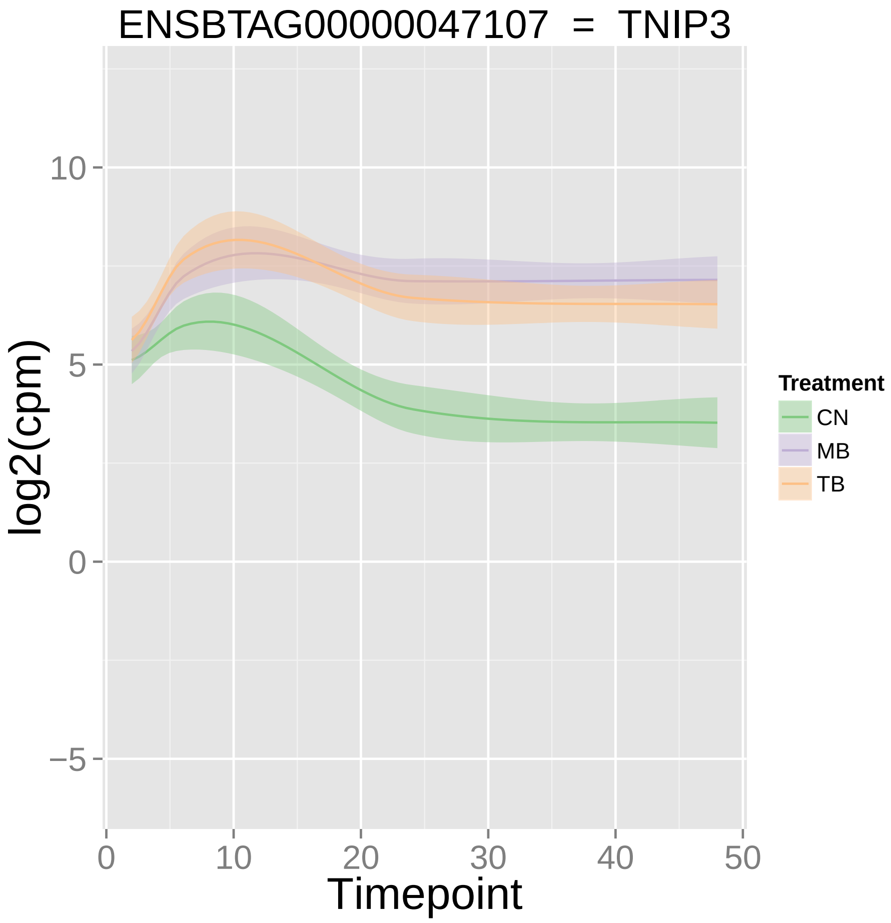

Note that `Timepoint` is another column of `pData(targets)`. that encodes a numeric vector, as opposed to the column named `Time`, encoding a factor. This difference enables the plotting function to respect the relative distance between the time-points for an output more representative of the actual time-scale.

To investigate the impact of other factors on the expression level of the same gene, users are encouraged to use the `f` and `x_var` arguments to specify alternate grouping factor and X variable, respectively. Note that the `geom_smooth` of the *ggplot2* package may fail if a minimal number of replicates is not available to calculate proper confidence intervals. In such cases, it is recommended to use the function `expression_profiles` described in section 3.9.

Here is another valid example separating samples by the factor `Animal` on the X axis and summarising all time-points in a confidence 95% confidence interval on the Y-axis:

```
> expression_plot(
+     gene_id = "ENSBTAG00000047107", result = BP.5, eSet=AlvMac,
+     x_var = "Animal", title.size=1.5, axis.text.angle=90,
+     legend.title.size=10, legend.text.size=10, legend.key.size=15)
```

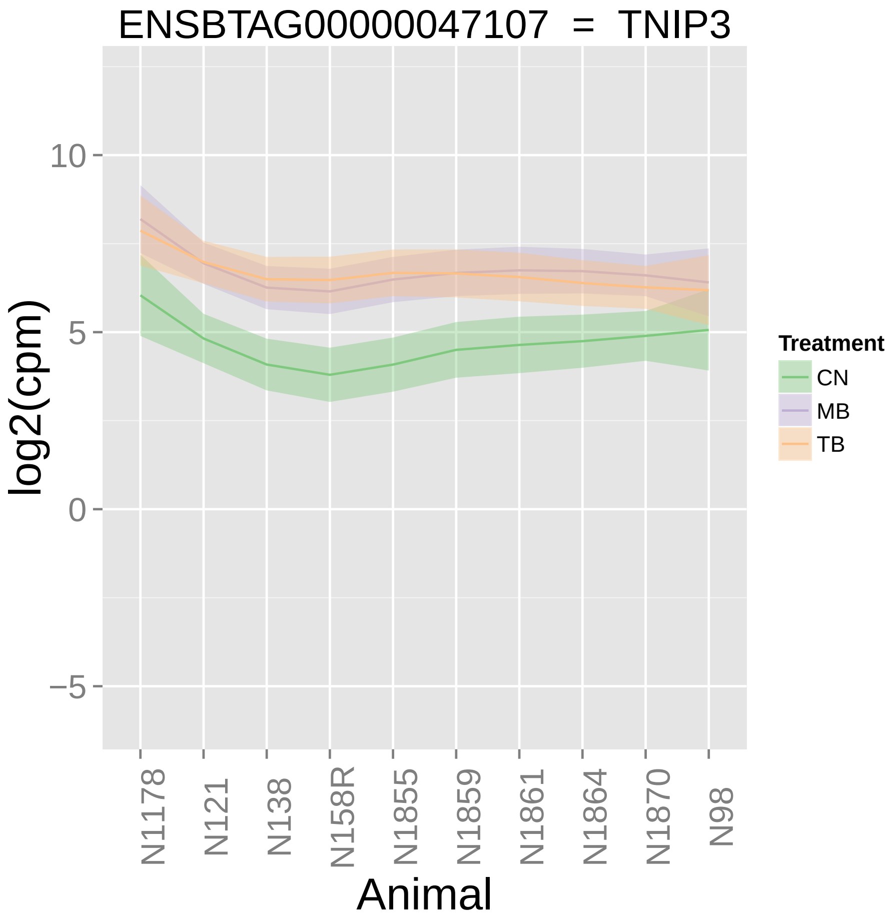

3.8.2 Using the associated gene name

It is also possible to visualise the expression profile of genes from their associated gene name if any. This is a more human-friendly version of the function presented in the previous subsection:

```
> expression_plot_symbol(
+     gene_symbol = "BIKBA", result = BP.5, eSet=AlvMac,
+     x_var = "Timepoint", title.size=1.5,
+     legend.title.size=10, legend.text.size=10, legend.key.size=15)
```

However, the benefits of this feature are balanced by the fact that genes lacking an associated gene name cannot be visualised in this manner, and that some gene symbols are associated with multiple Ensembl gene identifiers and probesets (e.g. 'RPL36A'). In the latter case, we turned the ambiguity into an additional useful feature: a lattice is created, and each of the multiple features associated with the given gene symbol are plotted simultaneously in the lattice. Subsequently, each of the sub-figures plotted may be re-plotted by itself using the `index` argument as indicated in the accompanying message printed in the *R* console:

```
> expression_plot_symbol(
+     gene_symbol = "RPL36A", result = AlvMac_results, eSet=AlvMac,
+     x_var = "Timepoint", title.size=1.5,
+     legend.title.size=10, legend.text.size=10, legend.key.size=15)
```

```
Fetching feature identifier(s) annotated to RPL36A ...
Multiple gene ids found for RPL36A
Indices are:
[1] "ENSBTAG00000025564" "ENSBTAG00000019253" "ENSBTAG00000027610"
Use argument 'index=1' to plot the first gene id alone, and so on.
Plotting ENSBTAG00000025564
```

```
Plotting ENSBTAG00000019253
Plotting ENSBTAG00000027610
[1] "ENSBTAG00000025564" "ENSBTAG00000019253" "ENSBTAG00000027610"
```

3.9 Expression profile of a gene by individual sample series

3.9.1 Using the unique feature identifier

It may be useful to track and visualise the expression profile of genes in each individual sample series, rather than their average. This could help identify outliers within sample groups, or visually compare paired samples, for instance.

In the `AlvMac` dataset, samples from each of the animals were subjected to all three treatments in parallel (i.e. paired samples). In the figure below, a sample series is defined by a given `Animal` and a given `Treatment`. Each sample series is then tracked over time, and coloured according to the `Treatment` factor (default, factor stored in `raw_results$factor`):

```
> AlvMac$Animal.Treatment <- paste(AlvMac$Animal, AlvMac$Treatment, sep="_")
> expression_profiles(
+     gene_id = "ENSBTAG00000047107", result = AlvMac_results,
+     eSet=AlvMac, x_var = "Timepoint", line.size=1,
+     seriesF="Animal.Treatment", linetypeF="Animal",
+     legend.title.size=10, legend.text.size=10,
+     legend.key.size=15)
```

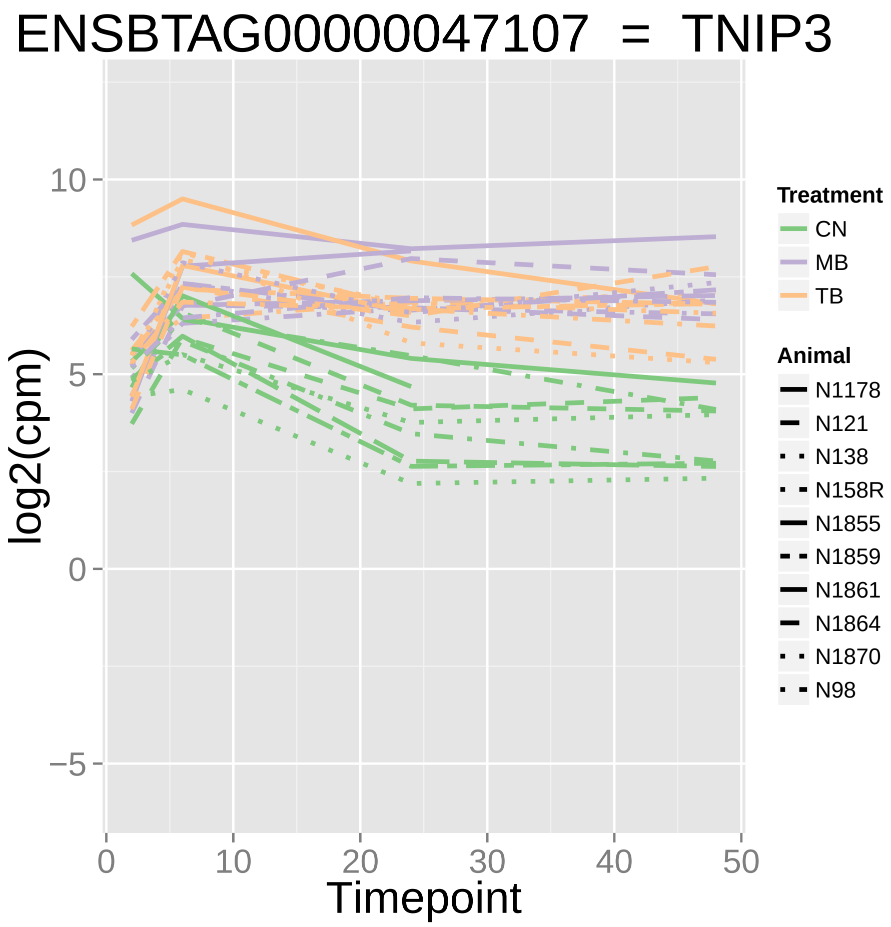

In the figure above, the `linetypeF` helps to highlight samples from an animal which start at unusually high expression values, while those samples progressively return to expression values similar to other samples in their respective treatment groups.

If omitted, the `linetypeF` argument will mirror the `colourF`, which can be useful for colour-blind people. Alternatively, a single line-type can be applied to all groups using the `lty` argument as follows:

```
> expression_profiles(
+     gene_id = "ENSBTAG00000047107", result = AlvMac_results,
+     eSet=AlvMac, x_var = "Timepoint",
+     lty=rep(1,10), # use line-type 1 for all 10 groups
+     seriesF="Animal.Treatment", linetypeF="Animal",
+     legend.title.size=10, legend.text.size=10,
+     legend.key.size=15, line.size=1)
```

3.9.2 Using the associated gene name

Similarly to the `expression_plot` function, an alternative was implemented to use gene names instead of feature identifiers. An example:

```
> expression_profiles_symbol(
+     gene_symbol="TNIP3", result = AlvMac_results,
+     x_var = "Timepoint", linetypeF="Animal", line.size=1,
+     eSet=AlvMac, lty=rep(1,10), seriesF="Animal.Treatment",
+     title.size=1.5, legend.title.size=10, legend.text.size=10,
+     legend.key.size=15)
```

3.10 Comparison of univariate effects on gene expression

While the analysis is restricted to the evaluation of a single factor, it can be helpful to compare the relative impact of all known factors present in the the accompanying `phenoData` on the gene expression in the different groups of samples.

In other words, given a GO term identifier this feature will generate a plot for each associated gene, where the mean (default; can be changed) expression level will be computed for each level of each factor and compared to one another:

```
> plot_design(
+     go_id = "GO:0034134", result = BP.5, eSet=AlvMac,
+     ask = FALSE, factors = c("Animal", "Treatment", "Time", "Group"),
+     main.Lsplit=30)
```

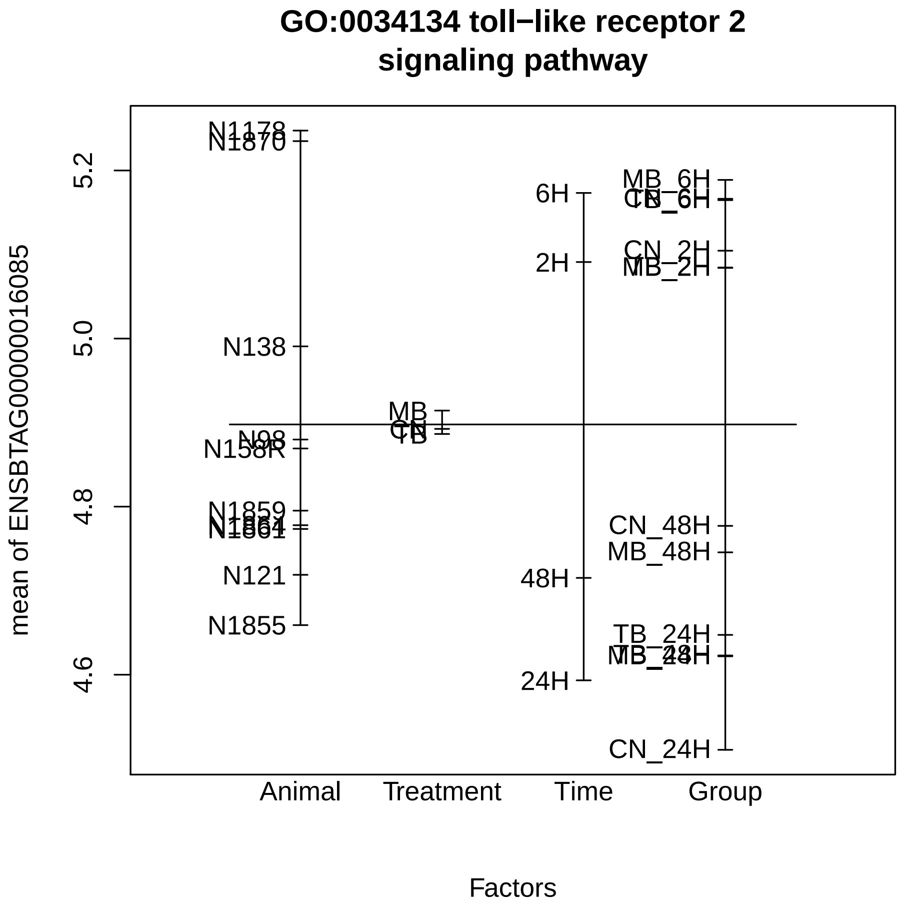

4 Additional controls and advanced functions

4.1 Custom annotations

To enable all downstream filtering and visualisation features, the `GO_analyse` function uses three types of annotations:

- `GO_genes`: Mapping of gene feature identifiers to gene ontology identifiers.
- `all_genes`: Annotations of gene feature identifiers.
- `all_GO`: Annotations of gene ontology identifiers.

The use of custom annotations has several advantages:

- **Traceability and reproducibility:** the Ensembl annotations are updated on a regular basis. A local copy of annotations allows use of archived annotation releases. In the absence of local annotations, `GO_analyse()` systematically connects to the latest (i.e. current) Ensembl release.
- **Speed:** Providing custom annotations will skip calls to the Ensembl server, significantly reducing the runtime of the `GO_analyse` function.
- **Autonomy from web-services:** occasionally the Ensembl server may be unavailable (e.g. maintenance, internet connection). A local copy of annotations allows to work independently from any web-service.
- **Alternative annotations:** Users working with gene feature identifiers not supported in the Ensembl annotations (e.g. species, or platforms), or comparing panels of biomarkers instead of GO terms for instance, may provide their own custom annotations to enable the ranking and visualisation of their data using *GOexpress*.

4.1.1 Generating custom annotations

Using the Ensembl release 75, we show below the code used to retrieve annotations for *Bos taurus* Ensembl gene identifiers:

```
> # Load the interface to BioMart databases
> library(biomaRt)
> # See available resources in Ensembl release 75
> listMarts(host='feb2014.archive.ensembl.org')
> # Connect to the Ensembl Genes annotation release 75 for Bos taurus
> ensembl75 = useMart(
+     host='feb2014.archive.ensembl.org',
+     biomart='ENSEMBL_MART_ENSEMBL', dataset='btaurus_gene_ensembl')
> ## Download all the Ensembl gene annotations (no filtering)
> allgenes.Ensembl = getBM(
+     attributes=c('ensembl_gene_id', 'external_gene_id', 'description'),
+     mart=ensembl75)
> # Rename the gene identifier column to 'gene_id'
> # This allows GOexpress to treat microarray and RNA-seq data identically
> colnames(allgenes.Ensembl)[1] = 'gene_id'
> ## Download all the gene ontology annotations (no filtering)
> allGO.Ensembl = getBM(
+     attributes=c('go_id', 'name_1006', 'namespace_1003'),
+     mart=ensembl75)
> ## Download all the mapping between gene and gene ontology identifiers
> GOgenes.Ensembl = getBM(
+     attributes=c('ensembl_gene_id', 'go_id'),
+     mart=ensembl75)
> # Rename the gene identifier column to 'gene_id'
> colnames(GOgenes.Ensembl)[1] = 'gene_id'
> # Cleanup: remove some blank fields often found in both columns
> GOgenes.Ensembl = GOgenes.Ensembl[GOgenes.Ensembl$go_id != '',]
> GOgenes.Ensembl = GOgenes.Ensembl[GOgenes.Ensembl$gene_id != '',]
```

comment: The automated retrieval procedure retrieves all gene ontology annotations from the Ensembl server, inclusive of annotations not Inferred from Experiment (EXP). Users may consider filtering local annotations for desired GO Evidence code(s).

4.1.2 Using custom annotations

The annotations download above can then be saved in local R data files, and subsequently used to run entirely offline analyses of *ExpressionSet* objects with corresponding gene feature identifiers:

```
> # save each custom annotation to a R data file
> save(GOgenes.Ensembl, file='GOgenes.Ensembl75.rda')
> save(allGO.Ensembl, file='allGO.Ensembl75.rda')
> save(allgenes.Ensembl, file='allgenes.Ensembl75.rda')
> # Run an analysis using those local annotations
> GO_analyse(
+     eSet=AlvMac, f='Treatment',
+     GO_genes=GOgenes.Ensembl,
+     all_GO=allGO.Ensembl,
+     all_genes=allgenes.Ensembl)
```

Ideally, all three annotation objects should be provided, to enable all downstream features. A toy example for each type of custom annotations, ready for analysis, is provided with the package:

```
> data(AlvMac_GOgenes)
> data(AlvMac_allGO)
> data(AlvMac_allgenes)
```

warning: Critically, it is highly recommended to provide full genome annotations for the species of interest, including annotations for feature identifiers that are absent from the given ExpressionSet. As described in section 5.1, all genes present in the annotations will affect the scoring of gene ontologies, even if they are absent from the ExpressionSet.

4.2 Using subsets of samples

It may be desirable to rank genes and gene ontologies according to their capacity to cluster only specific subsets of samples, while visualising the expression data of all samples. Instead of creating two separate *ExpressionSet* objects (one containing all the samples to visualise, another one containing only the samples to analyse), a `subset` argument was added to most functions in the *GOexpress* package, allowing the use of a single *ExpressionSet* object from which the desired subset of samples is extracted at run time.

This argument takes a named list where names must be column names existing in `colnames(pData(eSet))`, and values must be vectors of values existing in the corresponding column of `pData(eSet)`. The original *ExpressionSet* will be left unchanged. An example:

```
> AlvMac_results <- GO_analyse(
+     eSet = AlvMac, f = "Treatment",
+     subset=list(
+         Time=c("6H","24H", "48H"),
+         Treatment=c("CN","MB"))
+     )
> expression_plot(
+     gene_id = "ENSBTAG00000047107", result = BP.5, eSet=AlvMac,
+     x_var = "Timepoint", title.size=1.5,
+     legend.title.size=10, legend.text.size=10, legend.key.size=15,
+     subset=list(Treatment=c("TB","MB"))
+     )
```

4.3 Overlapping genes between GO terms

Often, the top-ranked cellular functions identified by pathway analysis and gene-set enrichment analysis tools may significantly overlap each other in terms of genes, which limits the number of genes identified by those approaches and blurrs the precise cellular function represented by the core gene-set among a list of closely related yet different functions.

Typically, overlapping sets are representated as Venn diagrams. The example below will produce a Venn diagram of the gene-sets associated with the five top-ranked "Biological Process" ontologies, and display it on screen, as the filename argument is left to the default `NULL` value (if a filename is given, the diagram will be printed to a TIFF file , no matter the extension specified):

```
> overlap_GO(go_ids = head(BP.5$GO$go_id, n=5), result = BP.5, filename=NULL)
```

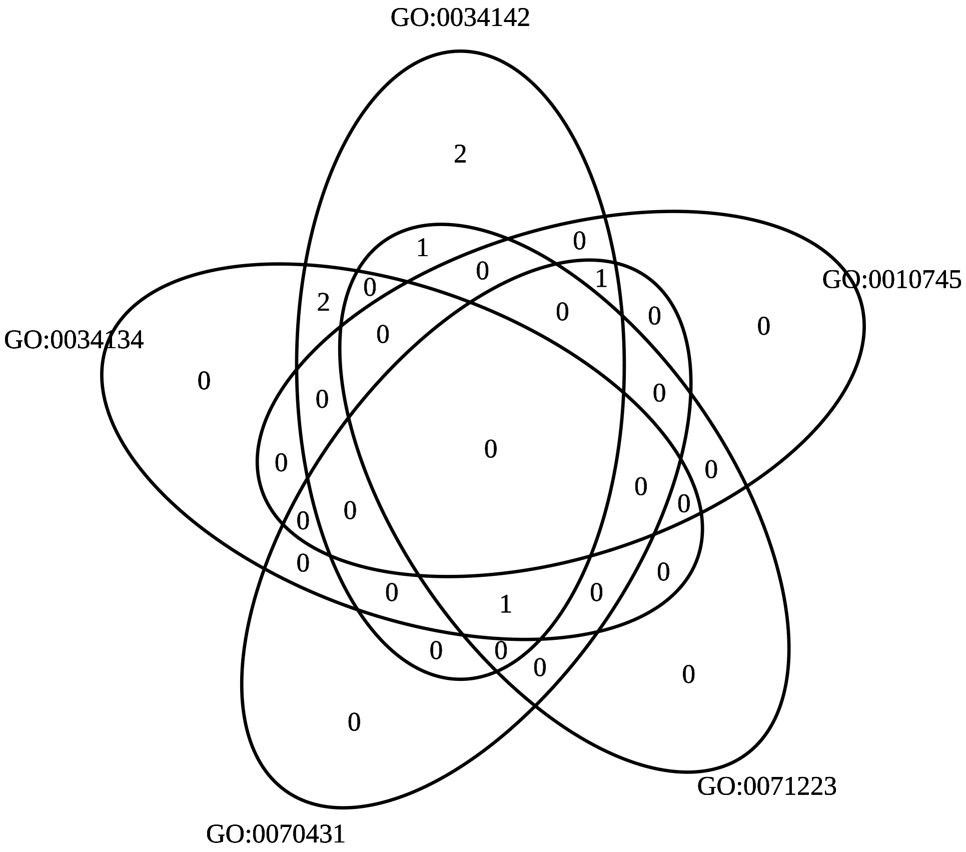

This function calls the `venn.diagram` function of the *VennDiagram* package, offering a high level of control on the resulting Venn diagram. A detailed description of the available arguments are accessible in the corresponding *R* documentation file.

4.4 Distribution of scores

Users might be interested in the general distribution of score and rank statistics produced by *GOexpress*. The distribution of scores may be represented as a histogram:

```
> hist_scores(result = BP.5, labels = TRUE)
```

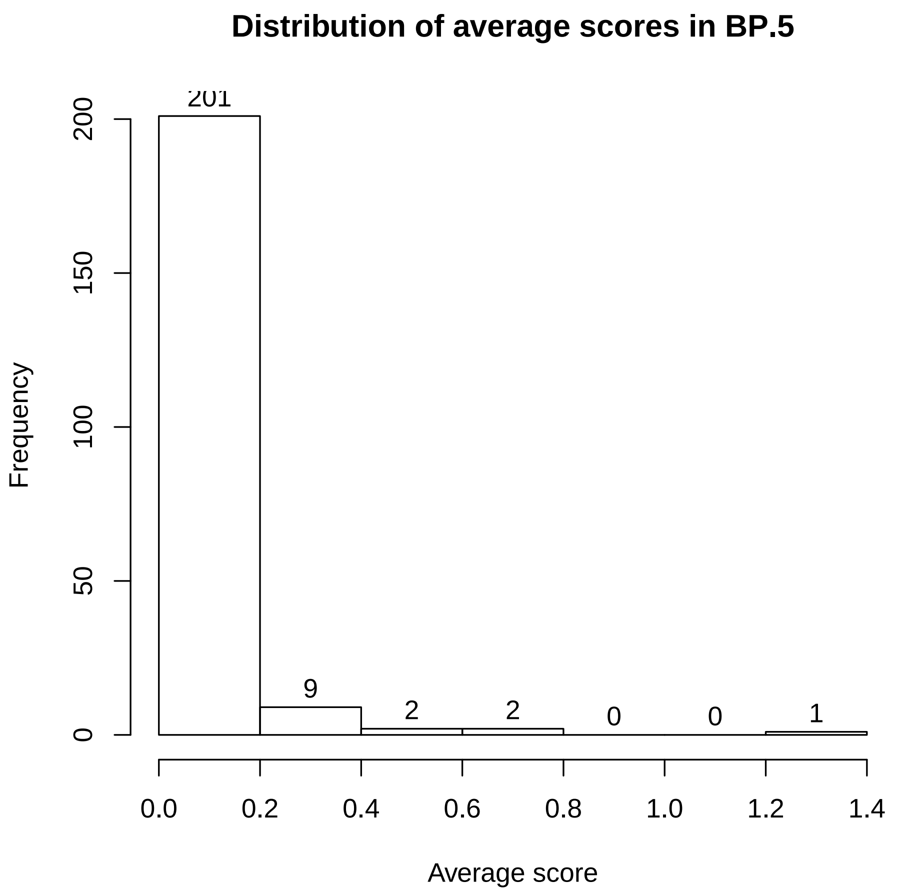

Alternatively, quantile values can be returned for default or customised percentiles:

```
> quantiles_scores(result = BP.5)

      90%       95%       99%     99.9%    99.99%
0.1479628 0.2207809 0.6464542 1.1998095 1.3150358
```

4.5 Reordering scoring tables

4.5.1 Reordering by score

While scores are more prone to extreme outlier values and may slightly fluctuate between multiple runs of the random forest algorithm; ranks of genes and subsequently average ranks of GO terms tend to be more stable and may be more reliable estimators of the importance of genes and cellular functions. Therefore, the default behaviour of *GOexpress* is to use the rank and average rank metrics to order genes and GO terms, respectively, in the returned score tables.

It is however possible to re-order the tables in the output variable according to the score metric (or revert back to the original one) as in the example below:

```
> BP.5.byScore <- rerank(result = BP.5, rank.by = "score")
```

4.5.2 Reordering by P-value

Additionally, it is possible to reorder GO terms by increasing P-value, provided those values were were computed using the `pValue_GO` function.

```
> BP.5.byPval <- rerank(result = BP.5, rank.by = "p.val")
```

4.5.3 Reordering and breaking ties

For instance, to rank GO terms by P-value, while breaking ties on their `ave_rank` value, one needs to first rank the object by `rank`, and rank the resulting object by `p.val`:

```
> BP.5.pVal_rank <- rerank(result = BP.5, rank.by = "rank")
> BP.5.pVal_rank <- rerank(result = BP.5.pVal_rank, rank.by = "p.val")
```

4.6 Subsetting an ExpressionSet to specific sample groups

While this feature exists, users may want to consider the newer section 4.2 describing the definition of a subset of samples from the given *ExpressionSet* on-the-fly without the need to create a new object containing the subsetted *ExpressionSet*.

It is straightforward to subset an *ExpressionSet* by extracting given columns (i.e. samples) and rows (i.e. gene features). Nevertheless, the *randomForest* package is quite sensitive to the definition of *R* factors; for instance, the `randomForest` function will crash if a factor is declared to have 3 levels (e.g. "A", "B", and "C"), while the *ExpressionSet* only contains samples for two of them (e.g. "A" and "B"). A simple fix is to update the known levels of the factor after having subsetted the *ExpressionSet*:

```
> pData(AlvMac) <- droplevels(pData(AlvMac))
```

comment: Note that this operation preserves the order of ordered factors.

Typically, subsetting an *ExpressionSet* by rows and columns does not automatically update the known levels of each factor to the remaining levels. The function `subEset` performs this additional task. The function takes a named list, where names must be column names from the `phenoData` slots and values must be present in the corresponding columns, and returns a subset of the original *ExpressionSet* including only the samples which match those values:

```
> subEset(
+     eSet=AlvMac, subset=list(
+     Time=c("2H","6H","24H"),
+     Treatment=c("CN","MB")))
```

5 Statistics

5.1 Overview

GOexpress was initially created from a set of gene-based, and later ontology-based, visualisation functions. Following the integration of those various plotting functions at the core of the *GOexpress* package, the need for a ranking of genes and GO terms soon became apparent in order to rapidly identify those with gene expression data best clustering the samples according to the experimental factor studied. A two-fold procedure was implemented:

1. Using the available expression data, each gene present in the dataset is scored, evaluating its ability to cluster the predefined groups of samples. Genes are ranked according to their respective score; ties are resolved by assigning the lowest rank R to all G genes, giving the rank G+n to the next gene(s). The genewise scoring functions implemented are described in the following subsections, and all were validated on in-house datasets cross-checked with comparable analytic pipelines (e.g. Ingenuity®Pathway Analysis, SIGORA)
2. Using the above ranks and scores, each GO term in the gene ontology annotations is assigned the mean score and the mean rank of all the genes associated with it in the gene-GO annotations. Genes present in the annotations but absent from the *ExpressioSet* are assigned a score of 0 (minimum valid score; indicates no power to discriminates the predefined groups of sample) and a rank equal to the number of genes present in the *ExpressioSet* plus one (worst rank, while preserving discrete continuity of the ranking).

5.2 Data-driven visualisation functions

Importantly, the statistics performed to rank GO terms and genes do *not* influence the behaviour of the subsequent plotting functions; heatmaps, dendrograms and gene expression profiles are purely driven by the expression data,

sample phenotype annotations provided and GO terms annotations, without any transformation or normalisation applied to the data. Therefore, users are encouraged to use and suggest alternative relevant scoring and ranking strategies, which could prioritise GO terms and genes in different ways. A current acknowledged bias is the higher scoring of GO terms associated with fewer genes, which is discussed in section 3.4.

5.3 Random Forest

We implemented the Random Forest framework to answer the question: "How well does each gene in the dataset cluster predefined groups of samples?". The random forest consists of multiple decision trees. Each tree is built based on a bootstrap sample (sample with replacement) of observations and a random sample of variables. The *randomForest* package first calculates the Gini index (Breiman et al, 1984) for each node in each tree. The Gini index is a measure of homogeneity from 0 (homogeneous) to 1 (heterogeneous). The decrease in the Gini index resulting from a split on a variable is then calculated for each node and averaged for each variable over all the trees in the model. The variable with the biggest mean decrease in the Gini index is then considered the most important. Technically, *GOexpress* extracts the `MeanDecreaseGini` value from the `importance` slot of the `randomForest` output and uses this value as the score for each gene.

A key feature of the Random Forest framework is the implicit handling of interactions between genes, given a sufficient number of trees generated and genes sampled. Indeed, at each node in the decision tree, genes are sampled from the expression data and tested for their individual capacity to improve the partitioning reached in the previous node. The larger the number of trees and genes sampled, the more complete the coverage of interactions will be.

5.4 One-way Analysis of Variance (ANOVA)

We implemented the ANOVA statistical framework to answer the question: "How different is the expression level of each gene in the dataset in the different groups of samples?". Given the expression level of a gene in all the samples, the one-way ANOVA determines the ratio of the variance between the groups compared to the variance within the groups, summarised as an F statistic that *GOexpress* uses as a score for each gene. Simply put, if samples from the same groups show gene expression values similar to each other while samples from different groups show different levels of expression, those genes will produce a higher score. This score cannot be less than 0 (variance between groups insignificant to variance within groups), while very large ratios can easily be reached for genes markedly different between groups.

Contrary to the random forest framework, the Analysis of Variance makes important assumptions on the data: namely, the independence of observations, the normality of residuals, and the equality of variances in all groups. While the former two are the responsibility of the user to verify, the latter is taken care of by *GOexpress*. Indeed, the `oneway.test` function of the package *stats* is used with parameter `var.equal` set to to `FALSE`. While this reduces the sensitivity of the test, all genes are affected by this correction based on the relative amount of variance in the different predefined groups of samples. Finally, it is once again important to note that the one-way ANOVA only evaluates univariate changes, while the random forest framework implicitely allows for interactions between genes.

6 Integration with other packages

6.1 shiny

Shiny is an *R* package that makes it easy to build interactive web applications (apps) straight from *R* as shown in Figure 1. More information is available at `http://shiny.rstudio.com/`.

7 Notes

7.1 Authors' contributions

Conception and development of the *GOexpress* package was carried out by KR-A with contributions by PAM, under the supervision of SVG and DEM. Experimental data used for testing was generated and analysed with the help of

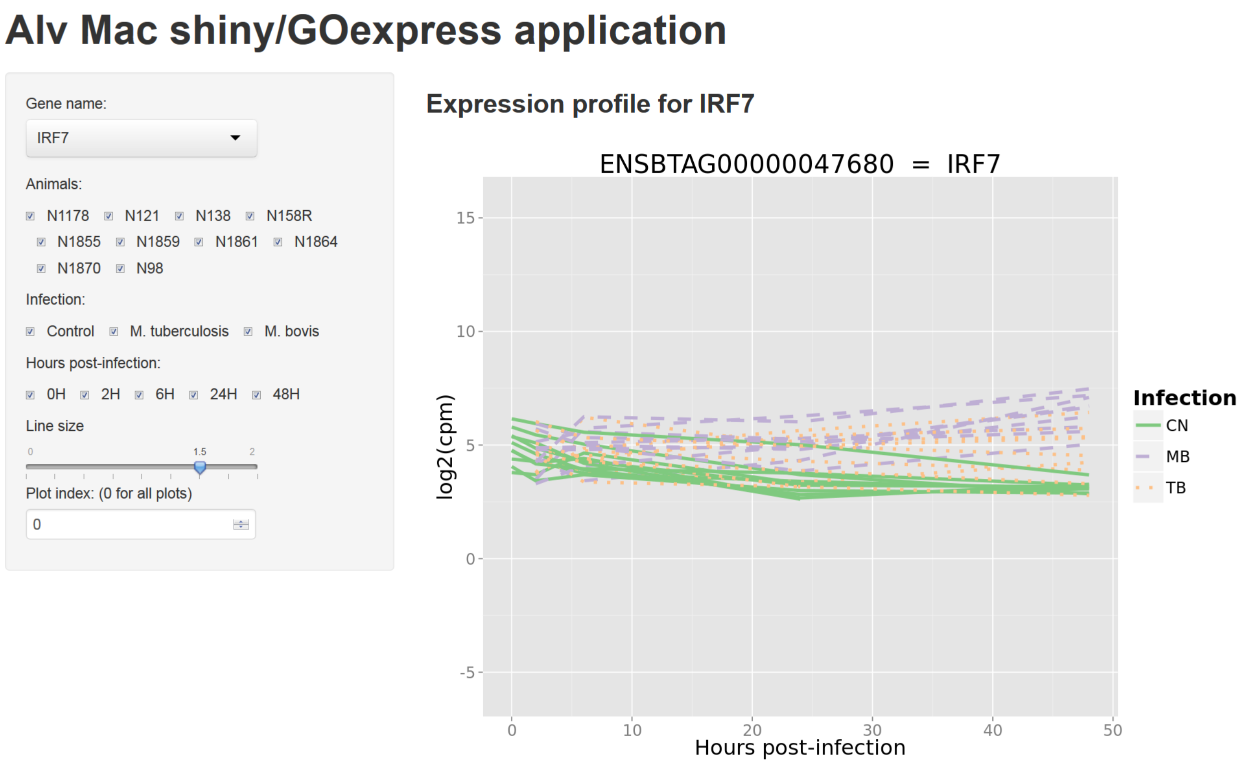

Figure 1: **Shiny app.** This simple application allows visualisation of genes using the *GOexpress* `expression_profiles_symbol` function. An online interactive version was made available at: `https://kevinrue.shinyapps.io/alvmac/`

DAM and NC. Integration of the random forest statistical frameworks was advised by BH and ACP. This User's Guide was prepared by KR-A, and edited by PAM, BH, DAM, NCN, ACP, SVG, and DEM.

7.2 Acknowledgments

Since the early beginning, *GOexpress* has grown from constructive feedback, and I would like to thank a number of colleagues and scientists from all backgrounds who contributed each in their own way to the present version and features of the package. Special thanks to Dr. Paul Cormican, Simone Coughlan, Dr. Karsten Hokamp, and an unknown reviewer for feedback leading to some features. Sincere thanks to Dr. Kate Killick for testing on human data and feedback. My thanks to the *Bioconductor* staff and in particular to Hervé Pagès for the helpful feedback which improved the standards of the code and documentation. Last but not least, thanks to the University College Dublin "OpenSequencing" group, the "Virtual Institute of Bioinformatics and Evolution" (VIBE), and the "UCD PhD Symposium in Computational Biology & Innovation" where I first presented raw versions of *GOexpress* and received valuable feedback and advice in return, underlying a significant number of updated and new features.

7.3 Session information

```
> sessionInfo()
```

```
R version 3.2.0 (2015-04-16)
Platform: x86_64-unknown-linux-gnu (64-bit)
Running under: Ubuntu 14.04.2 LTS

locale:
 [1] LC_CTYPE=en_US.UTF-8       LC_NUMERIC=C               LC_TIME=en_US.UTF-8
 [4] LC_COLLATE=C               LC_MONETARY=en_US.UTF-8    LC_MESSAGES=en_US.UTF-8
 [7] LC_PAPER=en_US.UTF-8       LC_NAME=C                  LC_ADDRESS=C
```

```
[10] LC_TELEPHONE=C             LC_MEASUREMENT=en_US.UTF-8 LC_IDENTIFICATION=C

attached base packages:
[1] parallel  grid      stats     graphics  grDevices utils     datasets  methods
[9] base

other attached packages:
[1] GOexpress_1.2.1     VennDiagram_1.6.9   Biobase_2.28.0      BiocGenerics_0.14.0

loaded via a namespace (and not attached):
 [1] Rcpp_0.11.5         AnnotationDbi_1.30.0 MASS_7.3-40          IRanges_2.2.0
 [5] munsell_0.4.2       colorspace_1.2-6     stringr_0.6.2        GenomeInfoDb_1.4.0
 [9] plyr_1.8.1          caTools_1.17.1       tools_3.2.0          gtable_0.1.2
[13] KernSmooth_2.23-14  DBI_0.3.1            gtools_3.4.2         randomForest_4.6-10
[17] digest_0.6.8        RColorBrewer_1.1-2   reshape2_1.4.1       ggplot2_1.0.1
[21] S4Vectors_0.6.0     bitops_1.0-6         RCurl_1.95-4.5       biomaRt_2.24.0
[25] RSQLite_1.0.0       labeling_0.3         gdata_2.13.3         gplots_2.16.0
[29] scales_0.2.4        stats4_3.2.0         XML_3.98-1.1         BiocStyle_1.6.0
[33] proto_0.3-10
```
