# Supplementary figures and images for: GOexpress: an R/Bioconductor package for the identification and visualisation of robust gene ontology signatures through supervised learning of gene expression data
Source: BMC Bioinformatics. 2016 Mar 11;17:126. doi: 10.1186/s12859-016-0971-3 (PMC4788925; doi:10.1186/s12859-016-0971-3)

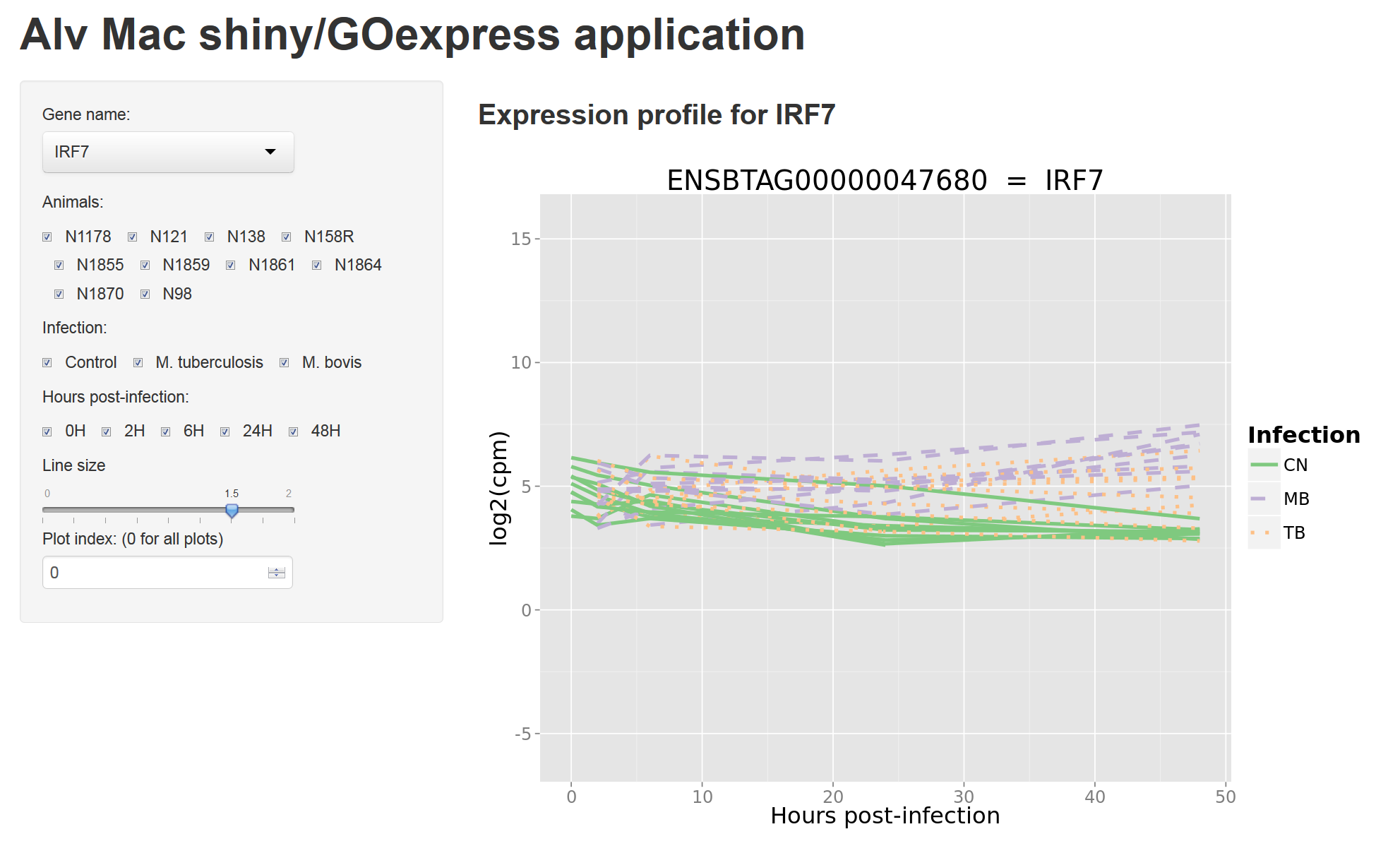

Supplement: Additional file 2: — Package source code for GOexpress release 1.2.1. (GZ 2281 kb) [file 12859_2016_971_MOESM2_ESM.gz › GOexpress/vignettes/images/shiny_screenshot.png]

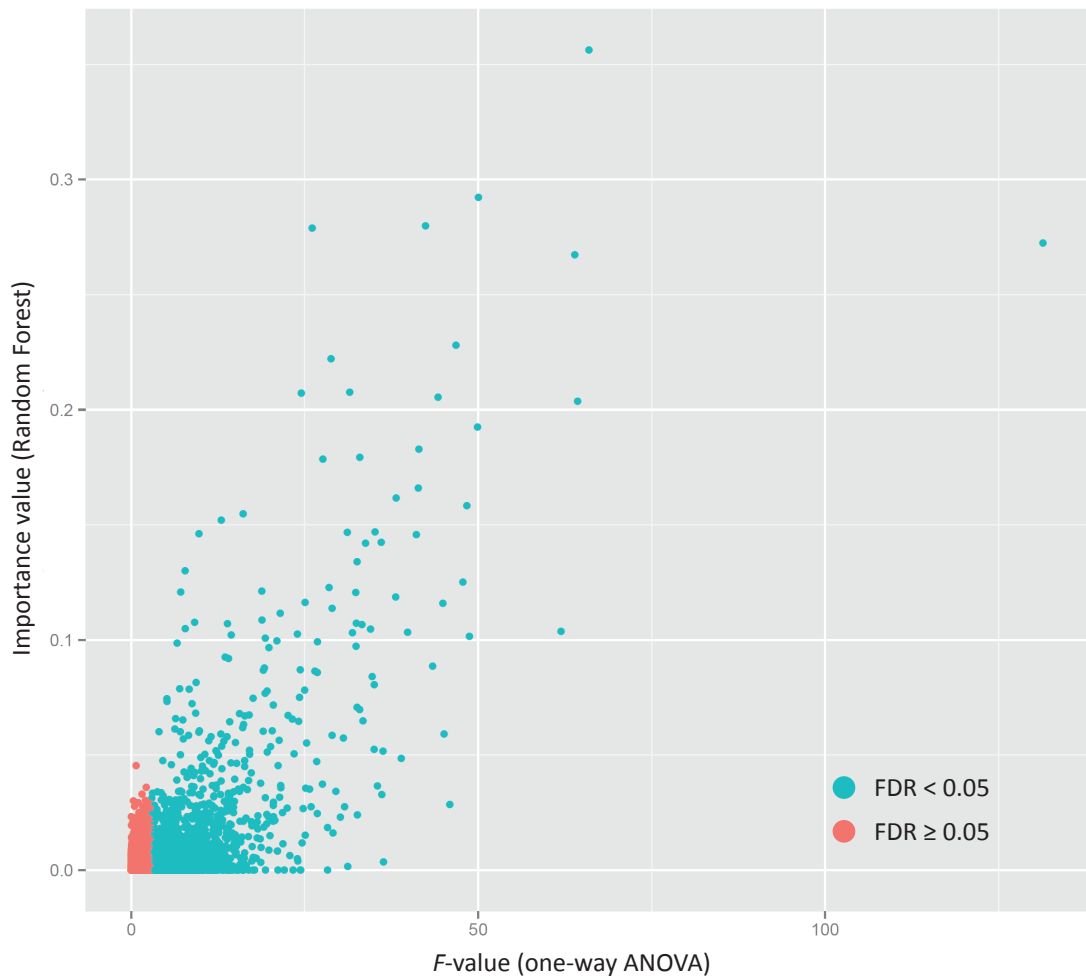

Supplement: Additional file 6: — Comparison of the importance score from the random forest algorithm (i.e., the mean decrease in Gini index) to the F-ratio from a one-way ANOVA. Probesets with large importance score (i.e., good classifiers) generally show a high F-ratio, indicative of high variance among the means of each group compared to the variance within the samples. (PDF 1215 kb) [file 12859_2016_971_MOESM6_ESM.pdf]
