# Supplementary material for: GOexpress: an R/Bioconductor package for the identification and visualisation of robust gene ontology signatures through supervised learning of gene expression data
Source: BMC Bioinformatics. 2016 Mar 11;17:126. doi: 10.1186/s12859-016-0971-3 (PMC4788925; doi:10.1186/s12859-016-0971-3)
Supplement: Additional file 3: — Pseudocode calculating the rank of GO terms and average score from the rank of gene features, shown in Fig. 2 and Additional file 7. (DOCX 15 kb) [file 12859_2016_971_MOESM3_ESM.docx]

Pseudocode 1: Algorithm to calculate the GO average rank (and average score) from the rank (and score) of annotated features.

1. Rank all features by decreasing score. Break ties by assigning the same minimal rank R to all F features, continuing with the rank R+F for the next feature(s).
2. For each GO term, fetch the list of annotated features.
   - Assign to each annotated feature absent from the ExpressionSet:
     - Score <- 0
     - Rank <- N_features_(ExpressionSet) + 1
   - Calculate for each GO term:
     - ave_Score <- Average score of annotated features
     - ave_rank <- rank of annotated features

N_features_: Number of features (*i.e.*, rows)
